# Combined Effects of Exercise and Phytoanabolic Extracts in Castrated Male and Female Mice

**DOI:** 10.3390/nu13041177

**Published:** 2021-04-02

**Authors:** Jerônimo P. Martins, Lucia C. Silva, Matheus S. Nunes, Gabriel Rübensam, Jarbas R. Oliveira, Rodrigo B. M. Silva, Maria M. Campos

**Affiliations:** 1 Programa de Pós-Graduação em Medicina e Ciências da Saúde, Escola de Medicina, Pontifícia Universidade Católica do Rio Grande do Sul, Porto Alegre, RS 90619-900, Brazil; jeronimo.martins@edu.pucrs.br (J.P.M.); jarbas@pucrs.br (J.R.O.); 2 Centro de Pesquisas em Toxicologia e Farmacologia, Escola de Ciências da Saúde e da Vida, Pontifícia Universidade Católica do Rio Grande do Sul, Porto Alegre, RS 90619-900, Brazil; lucia.silva.001@acad.pucrs.br (L.C.S.); matheus.nunes.002@acad.pucrs.br (M.S.N.); gabriel.rubensam@pucrs.br (G.R.); rodrigo.braccinims@gmail.com (R.B.M.S.); 3 Curso de Graduação em Fisioterapia, Escola de Ciências da Saúde e da Vida, Pontifícia Universidade Católica do Rio Grande do Sul, Porto Alegre, RS 90619-900, Brazil; 4 Curso de Graduação em Odontologia, Escola de Ciências da Saúde e da Vida, Pontifícia Universidade Católica do Rio Grande do Sul, Porto Alegre, RS 90619-900, Brazil; 5 Programa de Pós-Graduação em Odontologia, Escola de Ciências da Saúde e da Vida, Pontifícia Universidade Católica do Rio Grande do Sul, Porto Alegre, RS 90619-900, Brazil

**Keywords:** ergogenic phytotherapics, *Ajuga turkestanica*, *Eurycoma longifolia*, *Urtica dioica*, resistance exercise, ovariectomy, orchiectomy, sarcopenia, aging, mice

## Abstract

Dry extracts from the Eurasian plants, *Ajuga turkestanica*, *Eurycoma longifolia*, and *Urtica dioica* have been used as anabolic supplements, despite the limited scientific data on these effects. To assess their actions on early sarcopenia signs, male and female castrated mice were supplemented with lyophilized extracts of the three plants, isolated or in association (named TLU), and submitted to resistance exercise. Ovariectomy (OVX) led to body weight increase and non-high-density cholesterol (HDL) cholesterol elevation, which had been restored by exercise plus *U. dioica* extract, or by exercise and TLU, respectively. Orchiectomy (ORX) caused skeletal muscle weight loss, accompanied by increased adiposity, being the latter parameter reduced by exercise plus *E. longifolia* or *U. dioica* extracts. General physical activity was improved by exercise plus herbal extracts in either OVX or ORX animals. Exercise combined with TLU improved resistance to fatigue in OVX animals, though *A. turkestanica* enhanced the grip strength in ORX mice. *E. longifolia* or TLU also reduced the ladder climbing time in ORX mice. Resistance exercise plus herbal extracts partly altered gastrocnemius fiber size frequencies in OVX or ORX mice. We provide novel data that tested ergogenic extracts, when combined with resistance exercise, improved early sarcopenia alterations in castrated male and female mice.

## 1. Introduction

Declining of hormonal levels in aging is a predictor factor for the onset of skeletal muscle loss and related diseases, such as sarcopenia [1,2,3,4]. Sarcopenia is characterized by a progressive reduction in muscle quantity and quality, leading to impaired movement, lessened strength, with an increased risk of injuries secondary to falls, being often associated with frailty [5,6]. Nutritional intervention and physical exercise are valuable alternatives for prevention and management of sarcopenia [5,7,8]. Various drugs and supplements have emerged to treat sarcopenia, such as antioxidant supplementation, vitamin D, ursolic acid, angiotensin-converting enzyme inhibitors, melatonin, ghrelin, dehydroepiandrosterone (DHEA), and selective androgen receptor modulators (SARMS) [9,10,11,12,13,14]. Current treatments are focused on hormone replacement therapy, which might display adverse effects including exacerbation of sleep apnea, delayed wound healing, gynecomastia, increased volume of red blood cells, higher risk of cardiovascular diseases and cancer, besides virilization in women [15,16].

Phytotherapy represents an attractive option to treat several diseases and has been used for sarcopenia prevention and maintenance of hormonal levels [17,18]. Herbal medicine involves the use of the plant alone or in combination with other plants that have complementary properties. The association of selected herbal extracts has been employed as supplements to increase muscle mass gain, based on their folk use as anabolic agents. Particularly, dry extracts obtained from the Eurasian plants *Ajuga turkestanica*, *Eurycoma longifolia*, and *Urtica dioica* have been used for this end worldwide. However, there is limited scientific evidence about their efficacy and safety, and a very few studies have investigated their effects on sarcopenia.

*A. turkestanica*, a plant native from Uzbekistan, is rich in ecdysteroids, including turkesterone and 20-hydroxyecdysone (20-HE). It has been used for its benefits on muscle strength, muscle pain, and heart protection [19]. A study conducted by Isenmann et al. [20] investigated the effects of a commercial product containing 100 mg of ecdysteroids on strength and muscle mass of athletes. As evidenced, there was an increase in muscle mass, and in the concentration of serum insulin growth factor-1 (IGF-1), without changes in the concentrations of luteinizing hormone, testosterone, or estradiol [20]. Noteworthy, because of the hormone-related effects, this substance became part of the monitoring program of the World Anti-Doping Agency (WADA) in 2020 for professional athletes, inside and outside competitions [21].

*E. longifolia* Jack, also known as Malaysian Ginseng or Tongkat Ali, is commonly found in Southeast Asia. The plant is rich in several classes of bioactive compounds, such as eurycomanone, eurycomanol, 13α(21)-epoxueurycomanone, and 13α,21-dihydroeurycomanone [22,23]. A standardized extract of *E. longifolia* showed anti-adipogenic effects in animals [24] and improved several immunological parameters in humans [25]. Clinical studies have shown favorable results in the prevention of osteopenia in men and postmenopausal women [26]. In rats, *E. longifolia* led to increased serum concentrations of calcium, phosphate, and alkaline phosphatase, possibly through mechanisms related to hormonal modulation [27]. The root extract showed positive effects in increasing the sexual activity in a pre-clinical study [22], and it was able to rise hormone levels in animal [27] and clinical studies [28]. Though, another clinical study detected no benefit for the extract supplementation on resistance exercise outcomes [29].

The roots of the Eurasian plant *U. dioica* L., popularly known as stinging nettle, has been used to treat benign prostatic hyperplasia, for their anti-inflammatory effects and binding inhibition of sex hormone binding globulin (SHBG) to its receptor in prostate cells [30]. The compounds present in *U. dioica extract* include fatty acids, phenylpropanes, lignans, coumarins, triterpenes, ceramides, sterols, and lecithins [31,32]. Animals supplemented with *U. dioica* showed a reduction of adipose tissue accumulation, lower levels of fasting glucose and insulin, a decrease in HOMA-IR, and a reduction of liver triglycerides [33]. Compelling pre-clinical evidence revealed a hypoglycemic activity for the plant, and a meta-analysis carried out by Ziaie and collaborators (2019) corroborated the beneficial effects of *U. dioica* on glycemic levels. Pre-clinical in vitro and in vivo evidence also revealed the ability of *U. dioica* to inhibit 5-α-reductase activity, leading to an elevation of testosterone levels [34].

Considering the abovementioned evidence, the present study aimed to evaluate the anti-sarcopenic properties of *A. turkestanica*, *E. longifolia*, and *U. dioica* extracts, supplemented alone or in an association scheme, comparing the possible beneficial actions in castrated males and females submitted to resistance exercise.

## 2. Materials and Methods

### 2.1. Ethics and Experimental Animals

The experimental protocols used in this study followed the current Brazilian guidelines for the care and use of animals for scientific and didactic procedures, from the National Council for the Control of Animal Experimentation (MCTI-CONCEA, Brazil, 2016) [35], and were approved by the local Animal Ethics Committee (CEUA/PUCRS 8045/17). Male (weighing 18–30 g; total *n* = 200) and female (weighing 18–25 g; total *n* = 221) 12-week-old C57BL/6JUnib specific-pathogen-free mice were obtained from the Center for Experimental Biological Models (CeMBE/PUCRS). A sample size of 10–12 animals per group was determined a priori, based on previous publications using castration-related sarcopenia as the main outcome [9,36,37]. Exclusion criteria included animals that died after surgical procedures or gavage, and replacement was adopted to adjust the final sample size. The final N per group is indicated in the legend to figures and tables. Male and female mice were randomly distributed into different experimental cohorts, according to castration, climbing exercise and treatment with ergogenic plant extracts, as detailed in the next sections. A scheme showing the general study design is provided in the Supplementary Figure S1. All over the experimental time, animals were maintained in microisolator cages (maximum of 5 mice/cage), equipped with inlet/outlet air filters, under controlled temperature (22 ± 1 °C) and humidity (50–70%), under a light-dark cycle of 12 h (lights on at 7 a.m., lights off at 7 p.m.). The cages were covered with autoclaved wood chip bedding, and mice received standard pelleted chow and filtered water ad libitum. Behavioral and histological assessments were performed by an operator blinded to the experimental groups.

### 2.2. Surgical Castration

All the surgical procedures were performed under general anesthesia, with a mixture of xylazine (10 mg/kg) and ketamine (100 mg/kg), dosed by intraperitoneal (i.p.) route. Male and female mice were subjected to bilateral orchiectomy (ORX) or ovariectomy (OVX), respectively, according to the methodology described beforehand [9,38]. After a longitudinal incision in the dorsal skin and musculature, the testicles or the ovaries were exposed and a ligature was made for homeostasis, before surgically removing the gonads. The same procedure was carried out in the sham-operated groups (SHAM), but without ligature placement or organ removal. For pain control, the animals received acetaminophen (80 mg/kg) by oral route, for 48 h after surgery. Hereafter, the animal body weigh was monitored every week until the euthanasia.

Surgical castration was used as rodent models for inducing sarcopenia and sarcopenia-related functional changes, due to reduced levels of estrogen and testosterone, as it occurs in menopause and andropause [16,39,40,41,42].

### 2.3. Climbing Exercise Protocol

The resistance training was carried out by using the ladder climbing protocol. The procedures were initiated 8 weeks after ORX or OVX surgery. Sham-operated or castrated mice were subjected to a protocol of morning resistance exercise, three times a week, in a step ladder with 2-mm grids and one-meter height, with an inclination of 85 degrees. For adaptation, the animals were trained to climb the ladder for three days, one week before. On the first day, each animal was submitted to two series with three repetitions of climbing. On days 2 and 3, the training was performed in four series with three repetitions. For eight weeks, the sections of resistance exercise were performed by placing different weights in the base of the mouse tail with an adhesive tape. The initial load corresponded to 50% of the animal body weight, being progressively increased every seven days by 10%. Each section consisted of four series with 1-min resting, which were repeated three times in intervals of 2 min. The method was adapted from previous publications [43,44]. Sedentary groups of sham-operated or castrated mice were adapted in the same environment, but without any protocol of exercise. For animals submitted to resistance training, the ladder climbing time (s) was registered as the mean of three trials, as an additional measure resistance, at 16 weeks after castration (8 weeks after the onset of climbing exercise).

### 2.4. Treatment with Ergogenic Phytocompounds

Male and female mice from the different experimental groups, castrated or SHAM, sedentary or submitted to climbing exercise, were randomly distributed into five treatment groups that received: (i) vehicle (0.9% NaCl solution) 10 mL/kg; (ii) *Ajuga* turkestanica lyophilized extract (containing 2.5% of turkesterone) 50 mg/kg; (iii) *Eurycoma longifolia* lyophilized extract (containing eurycomanone) 200 mg/kg; (iv) *Urtica dioica* lyophilized extract 50 mg/kg (containing 0.82% of β-sitosterol); (v) a combination of the three extracts (named as TLU). The phytoanabolic extracts were administered daily by oral route, for eight weeks, beginning eight weeks after castration. The doses of the extracts were based on a series of previous publications showing efficacy and low toxicity in different experimental paradigms [34,45,46,47,48,49,50,51,52,53,54,55,56,57,58,59]. A scheme showing the timeline for the experimental protocols is provided in the Supplementary Figure S2. Within the figure and table legends, the following abbreviations have been used: SHAM, sham-operated; OVX, ovariectomized; ORX, orchiectomized; Exer, exercise (group submitted to exercise protocol); Sed, sedentary (that have not been submitted to exercise protocol) Turk, *Ajuga turkestanica*; Long, *Eurycoma longifolia*; Urt, *Urtica dioica*; TLU, the association of the three extracts.

### 2.5. Determination of Active Principles in Lyophilized Extracts

Samples were individually added in methanol (LiChrosolv, Merck, Darmstadt, Germany) at concentration of 50 mg/mL and agitated in an orbital agitator Mixer 099A (Glas-Col, LLC, Terre Haute, IN, USA) at 40 RPM, for 20 min, at room temperature (RT). Each mixture was incubated in an ultrasound bath T-14 (Thornton, São Paulo, Brazil) for 5 min, at RT and the dissolved phase was used for subsequent determinations.

Total content of phytosterols was determined by spectrophotometry, as previously described [60], using high-density cholesterol (HDL)-cholesterol (100 mg/L) as reference standard (Bioclin, Minas Gerais, Brazil) and expressing the results as milligram equivalents of HDL-cholesterol per gram of lyophilized extract (mgEq/g). Briefly aliquots of each sample were subsequently evaporated, resuspended in chloroform (Ensure, Merck, Germany), derivatized with Liebermann–Burchard reagent (LB), and read at 625 nm in a spectrophotometer (Spectramax M5, Molecular Devices, San Jose, CA, USA), using chloroform as analytical blank. LB was prepared by mixing 20 mL of ice cooled acetic anhydride (96%, J.T.Baker, Phillipsburg, NJ, USA) with 2 mL of sulfuric acid (98%, Merck, Germany).

Phytosterols were individually quantified by liquid chromatography with UV detection (LC-UV), using an Agilent LC system 1260 Infinity (Agilent Technologies, Santa Clara, CA, USA). Chromatographic separations were performed using a Zorbax Eclipse PLUS C18 RRHD 2.1 × 50 mm 1.8-µm column (Agilent, Paolo Alto, CA, USA), with 0.1% formic acid (eluent A) and 0.1% formic acid in methanol (eluent B) as the mobile phase, in gradient mode. The gradient was programmed to start with 20% of eluent B. After 2 min, eluent B was increased to 60%, remaining at this condition for 3 min. For column cleanup step, eluent B was increased to 100% for 7 min, and changed back to 20% to equilibrate the system before the next sample injection. The total time of analysis was 20 min. Ten microliters of samples were injected into the system, and the column temperature was maintained at 50 °C. The detector was operated simultaneously at 240 and 254 nm. HDL-cholesterol (Bioclin, Minas Gerais, Brazil) was used as the reference steroid for the calibration curve.

All quantified phytosterols were confirmed by liquid chromatography tandem mass spectrometry (LC-MS/MS), on an Agilent 6460 Triple Quad mass spectrometer coupled to Agilent 1290 Infinity chromatograph (Agilent, Paolo Alto, CA, USA). Phytosterol separations were performed by a similar chromatographic method as used for LC-UV analysis injecting; however, only five microliters of sample were used due to the method sensitivity. Analytes were ionized in an electrospray ionization (ESI) source and analyzed in multiple reaction monitoring mode. The ESI source was operated in positive ion mode, at 350 °C temperature, cone gas flow rate of 11 L/min, desolvation gas flow rate of 10 L/min, and a capillary volage of 3500V. The transitions (*m*/*z*) monitored were: eurycomanone (*m*/*z* 393.4 > 237.2 and 393.4 > 355.2); β-sitosterol (397.0 > 81.0 and 397.0 > 95.0); turkesterone (*m*/*z* 497.0 > 443.0 and 497.0 > 461.0).

### 2.6. Voluntary Locomotor Activity

Considering the motor deficits related to sarcopenia, the overall spontaneous locomotor activity of animals was assessed at 12 and 16 weeks after surgical procedures in the different experimental groups [61]. For this purpose, an automatic system comprised of an acrylic box (46 × 46 × 36 cm) with infrared sensors was used. The animals were placed in the center of the arena, with 1 min for habituation and 5 min for locomotor activity analysis. The total activity time (s), the traveled distance (cm), and the speed (mm/s) were registered. Movements were monitored on the x, y, and z-axes, which represent the height, width, and depth, respectively.

### 2.7. Rota-Rod Performance

The time until to fatigue was evaluated in the rota-rod apparatus, which consisted of a rod (3-cm in diameter) with five flanges, allowing the simultaneous evaluation of four mice. Mice were trained in the device 24 h before the experimental session. For training, the animals were placed in the device at a speed of 9 RPM until 60 s, and the procedure was repeated three times, with a 15-s interval. For the experimental sessions, mice were positioned in the equipment at an initial speed of 9 RPM, with an automatic gradual speed increase every 36 s, until reaching the maximal velocity of 40 RPM. The latency to fall (s) was registered as the mean of three trials, with a 15-s interval [62]. The evaluations were carried out at 12 and 16 weeks after surgical castration.

### 2.8. Grasping Test

This test was conducted to determine the grip strength of animals in the different experimental groups, at 12- and 16-weeks post-castration. After a gentle lifting by the tail, mice were allowed to grasp a grid positioned on an electronic balance. Animals were submitted to three trials, with a 15-s interval. The number (in grams) showed by the balance was instantly recorded, and a mean was calculated after the three trials to obtain the individual grasping strength.

### 2.9. Euthanasia and Sample Collection

One day after behavioral evaluations, mice were euthanized by deep isoflurane inhalation anesthesia. The blood was collected from the abdominal aorta for subsequent analysis. The following organs were sequentially collected and weighted (in g): gastrocnemius, tibialis anterior, soleus, subcutaneous adipose tissues, liver, kidney, bone, and brain.

### 2.10. Biochemical Analysis

The serum levels of triglycerides, total cholesterol, high-density cholesterol (HDL), non-HDL, aspartate transaminase (TGO) and alanine aminotransferase (TGP) were measured using commercial kits (Labtest, Lagoa Santa, Brazil).

### 2.11. Cytokine Determination

The levels of interleukin-1β (IL-1β), tumor necrosis factor (TNF), and interleukin-10 (IL-10) were measured in gastrocnemius muscle samples obtained from the different experimental groups. The cytokine levels were assessed by sandwich enzyme-linked immunosorbent assay using DuoSet kits according to the manufacturer’s instructions (R&D Systems, Minneapolis, MN, USA). The results are expressed in pg/mL.

### 2.12. Histological Analysis of Skeletal Muscle and Adipose Tissue

After registering the wet weight, the gastrocnemius and subcutaneous inguinal adipose tissue were processed for histological assessment. Tissues were fixed in 10% formaldehyde, during 24 h. Subsequently, the samples were included in paraffin and sectioned in 5-µm slices, for staining with hematoxylin and eosin. Images were captured using a Zeiss AxioImager M2 light microscope under 400× magnification (Carl Zeiss, Gottingen, Germany) and were analyzed using NIH ImageJ 1.36b Software. Adipocyte diameter was measured by using an automated tool, whereas the muscle fiber cross-sectional areas (CSA) were measured manually, using the free-hand function, after adjusting the image scale in µm^2^. The frequency size distribution of muscle or adipocyte areas were also determined. For histological analysis, 5–7 slides were evaluated from each group, in a blinded manner.

### 2.13. Statistical Analysis

Results are provided as the mean ± SEM. For time-course data, the areas under the curve (AUC) were calculated before statistical comparisons. The Shapiro–Wilk test was used to check data normality. Statistical analysis was performed by One-way ANOVA followed by Sidak post hoc test or by Two-way ANOVA followed by Uncorrected Fisher’s LSD. Statistical tests and design of graphs were held using GraphPad Software version 8.4.3 (GraphPad Software Inc., San Diego, CA, USA).

## 3. Results

### 3.1. Analysis of Bioactive Compounds in Lyophilized Extracts

**Figure 1:**
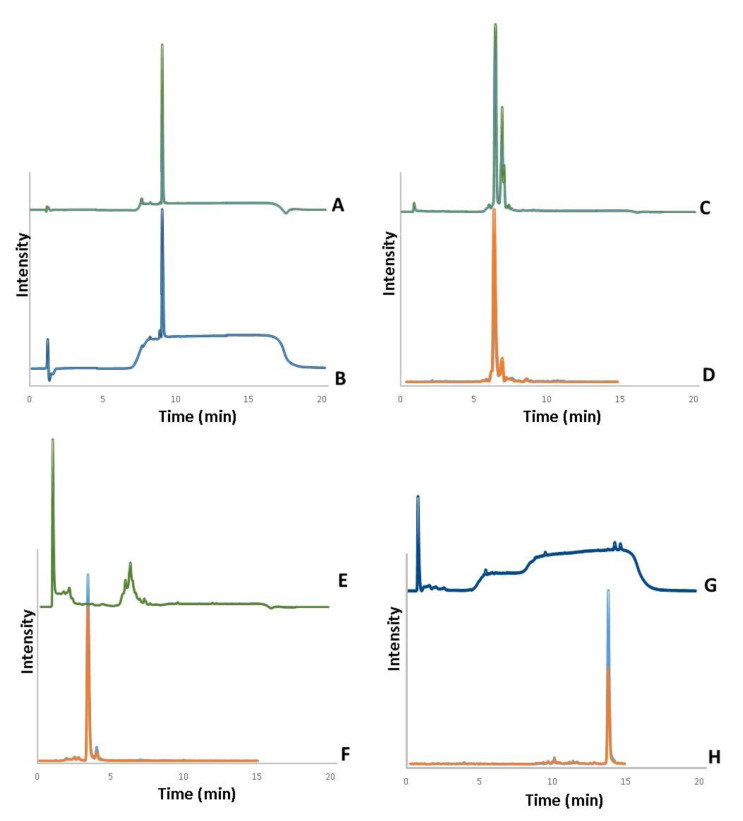
Identification of main active compounds in the ergogenic extracts. Cholesterol 50 mg/L (**A**) by LC-UV in 240 nm, (**B**) in 254 nm; Turkesterone (**C**) by LC-UV in 254 nm and (**D**) by LC-MS/MS with the fragments *m*/*z* 497.0 > 443.0 (light blue) and 497.0 > 461.0 (orange); Eurycomanone (**E**) by LC-UV in 254 nm and (**F**) by LC-MS/MS with the fragments *m*/*z* 391.5 > 251.1 (light blue) and 391.5 > 279.1 (orange); β-sitosterol (**G**) by LC-UV in 240 nm and (**H**) by LC-MS/MS with the fragments *m*/*z* 397.0 > 81.0 (light blue) and 397.0 > 95.0 (orange).

The analysis of *A. turkestanica* extract indicated a content of 7.8% regarding the total phytosterols (78.0 mgEq/g), containing 0.501 mg of turkesterone per gram, as determined by LC-UV (Figure 1C), and confirmed by LC-MS (Figure 1D). As for *E. longifolia* Jack, the extract presented 0.5% of total phytosterols (5.0 mgEq/g), with 0.009 mg of eurycomanone per gram (Figure 1E,F). The lyophilized extract of *U. dioica* presented 2.8% of total phytosterols (28.0 mgEq/g), which corresponded to 0.016 mg of β-sitosterol per gram (Figure 1G,H). HDL-cholesterol was used to determine total content of phytosterols by VIS and to estimate the concentration of each phytosterol by LC-UV (Figure 1A,B).

### 3.2. Castration Effects Prior to Exercise and Treatments

**Figure 2:**
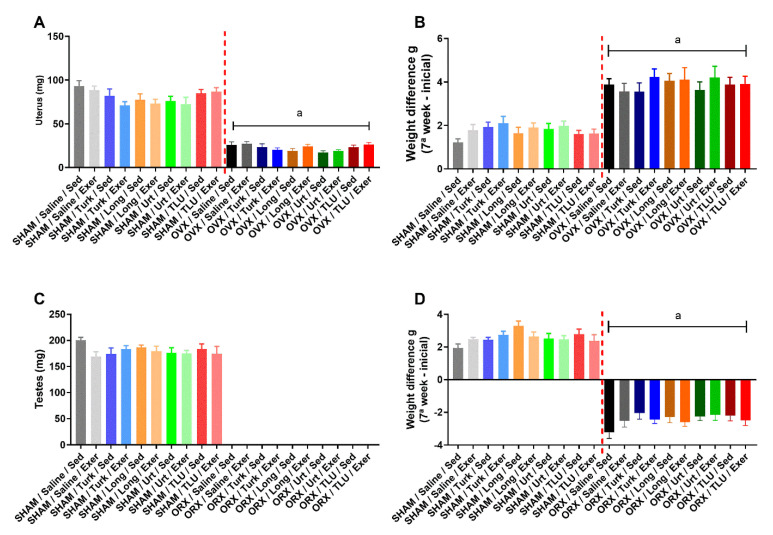
Effects of castration on uterus (**A**) and testes weight (**C**), 16 weeks after ovariectomy and orchiectomy, respectively, in comparison with sham-operated mice. Impacts of ovariectomy (**B**) and orchiectomy (**D**) on the body weight, 7 weeks after castration and before exercise and treatment onset. SHAM, sham-operated; OVX, ovariectomized; ORX, orchiectomized; Sed, sedentary; Exer, Exercise; Turk, Ajuga turkestanica; Long, Eurycoma longifolia; Urt, Urtica dioica; TLU, the association of the three extracts. Results are the mean ± SEM of 10 to 12 animals per group. ^a^ *p* < 0.05 significantly different compared to the sham-operated group (One-way ANOVA followed by Sidak post hoc test).

OVX resulted in a significant increase of body weight gain in comparison with the corresponding SHAM females, after seven weeks of surgery (Figure 2B). Differently, ORX led to body weight loss, compared with SHAM males (Figure 2D), as evaluated at the seventh week post-castration. Animals were distributed into sedentary or climbing exercise groups, and additional experimental treatment subgroups were created avoiding any significant differences when comparing the mean body weights, for either females (Figure 2B) or males (Figure 2D). Castration was confirmed by a marked reduction of uterus weight in females (Figure 2A) and the absence of testes in males (Figure 2C), as evidenced at the moment of euthanasia (16 weeks).

### 3.3. Changes in Body Weight after Exercise and Treatments

**Figure 3:**
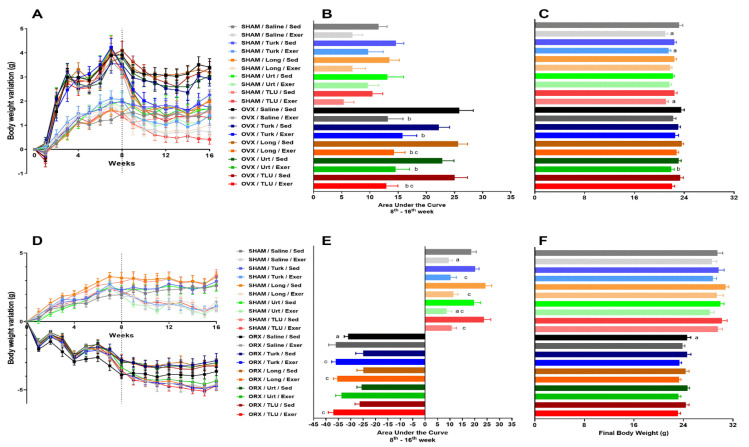
Changes in body weight in female (**A**) and male (**D**) mice, with treatments and exercise since castration until euthanasia. Area Under the Curve for the treatments and exercise period, from 8 to 16 weeks in females (**B**) and males (**E**). Final body weight of female (**C**) and male (**F**) mice. SHAM, sham-operated; OVX, ovariectomized; ORX, orchiectomized; Sed, sedentary; Exer, Exercise; Turk, Ajuga turkestanica; Long, Eurycoma longifolia; Urt, Urtica dioica; TLU, the association of the three extracts. Results are the mean ± SEM of 10 to 12 animals per group. ^a^ *p* < 0.05 significantly different compared with the sham-operated group treated with saline. ^b^ *p* < 0.05 significantly different compared with the castrated group treated with saline. ^c^ *p* < 0.05 significantly different comparing exercise to the respective sedentary group (One-way ANOVA followed by Sidak post hoc test).

The body weight was monitored weekly from the time of castration until euthanasia, at 16 weeks. Time-related body weight variations over the weeks are depicted in Figure 3A,D, for females and males, respectively. The area under the curve (AUC), from 8 to 16 weeks, was calculated to assess the effects of climbing exercise and ergogenic extracts in SHAM and castrated groups. OVX triggered a significant increase of AUC in saline-treated sedentary female mice, whereas the climbing exercise was able to significantly reduce the body weight gain in OVX animals, reaching similar levels as seen in SHAM sedentary animals, irrespective of treatment with the extracts. The exercise also led to diminished body weight gain in SHAM groups, although significant differences were not observed (Figure 3B). Alternatively, the resistance exercise significantly reduced the final body weight of saline-treated SHAM females, an effect that was mirrored in the exercise groups treated with *A. turkestanica* extract or TLU. Regarding the OVX animals, only the combination of exercise and *U. dioica* extract significantly reduced the final body weight (Figure 3C).

SHAM male mice submitted to climbing exercise displayed a decrease of AUC body weight gain, independent on the supplementation with any extracts. ORX sedentary males presented a marked loss of body weight gain, without any beneficial effect for resistance exercise in saline-treated animals. The treatment of sedentary ORX mice with the three extracts, alone or in combination, partially reversed the body weight loss induced by castration. The combination of ergogenic extracts with exercise failed to recover the body weight loss in ORX mice (Figure 3E). Sedentary ORX animals showed a significant reduction of final body weight, when compared with sedentary saline-treated SHAM males. Either exercise or ergogenic extracts failed to recover this parameter (Figure 3F). The mean values for initial, final, and final minus initial body weight for the different experimental groups are presented in Table 1 and Table 2, for females and males, respectively.

### 3.4. Combined Effects of Ergogenic Extracts and Exercise on Skeletal Muscle Weight

The skeletal muscle weight is an essential marker for defining muscle quantity. As indicated in Table 1, OVX did not evoke any significant alteration of gastrocnemius, tibial or soleus muscle wet weights, even when the summed weights of the three muscles were considered, in relation to the corresponding SHAM controls. For females, the treatment of sedentary OVX mice with *A. turkestanica* extract induced a significant reduction of summed wet weight of gastrocnemius, tibial and soleus, when compared with the respective saline-treated sedentary OVX group. The correction of muscle weight per body weight (in %) did not show any significant differences among the groups. Climbing exercise or supplementation with extracts failed to alter the wet weight of skeletal muscles in SHAM females (Table 1).

In males, castration significantly reduced the wet weight of the gastrocnemius, tibial and soleus muscles, which was confirmed when the total weight of the three muscles was determined. Nor exercise or the extracts significantly changed skeletal muscle mass in SHAM or ORX mice. As for the correction of muscle weight per body weight (in %), the association of climbing exercise with *U. dioica* extract increased this parameter in a significant manner (Table 2).

### 3.5. Adipose Depots and the Effects of Exercise and Ergogenic Extracts

In this study, the inguinal fat depots (iWAT) were evaluated as an indicative of white adipose tissue contents, whilst the interscapular fat (iBAT) was used to assess the presence of brown adipose tissue. In females, the wet weight of iWAT and iBAT, even after correction per body weight (in %), did not show any significant differences when comparing SHAM and OVX mice, regardless of exercise or administration of extracts (Table 1). As well, males did not exhibit any significant differences of iBAT (absolute weight or corrected values), when the effects of castration, resistance exercise or ergogenic extracts were evaluated (Table 2). Of note, ORX led to a marked increase of iWAT wet weight, when compared with the corresponding saline-treated SHAM group. This result was confirmed after correction of iWAT weight per body weight. ORX mice submitted to climbing exercise presented a trend toward a reduction of iWAT wet weight, but this effect was significant only in the exercise groups that had been treated with *E. longifolia* or *U. dioica* extracts. The beneficial effects of resistance exercise combined with *U. dioica* extract were also observed after analysis of iWAT weight corrected per body weight (in %) (Table 2).

### 3.6. Exercise and Treatment with Phytoanabolic Extracts and Their Effects on Functional Parameters

**Figure 4:**
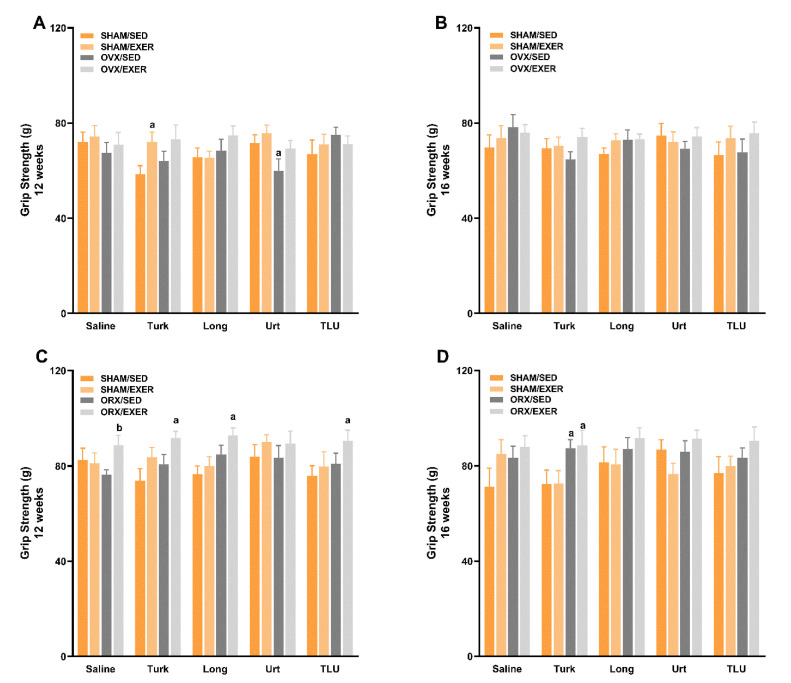
Grip strength assessed at 12 and 16 weeks in female ((**A**) and (**B**), respectively) and male ((**C**) and (**D**), respectively) mice. SHAM, sham-operated; OVX, ovariectomized; ORX, orchiectomized; Sed, sedentary; Exer, Exercise; Turk, Ajuga turkestanica; Long, Eurycoma longifolia; Urt, Urtica dioica; TLU, the association of the three extracts. Results are the mean ± SEM of 10 to 12 animals per group. ^a^ *p* < 0.05 significantly different compared with the sham-operated group. ^b^ *p* < 0.05 significantly different compared exercise to respective sedentary treatment (two-way ANOVA followed by Uncorrected Fisher’s LSD).

Reduced hormone levels and aging-related sarcopenia likely impair muscle strength, resistance to fatigue and spontaneous locomotor behavior. SHAM females submitted to climbing exercise and treated with *A. turkestanica* extract presented an increase of grip strength when compared with their sedentary counterparts, according to the evaluation at 12 weeks (i.e., 4 weeks after intervention onset). Alternatively, there was a reduction of grip strength in OVX sedentary mice that had been treated with *U. dioica*, at the same experimental time (Figure 4A). Neither of the female groups exhibited any significant difference of grip strength at 16 weeks (Figure 4B). At 12 weeks, the protocol of resistance exercise improved the grip strength of ORX mice in all of the extract-treated groups, except in the group that received *U. dioica* supplementation (Figure 4C). The treatment with *A. turkestanica* extract improved the grip strength of ORX mice at 16 weeks, regardless of exercise training (Figure 4D).

By using a protocol adapted to assess fatigue resistance in the rotarod apparatus, it was possible to observe that OVX led to a reduction of resistance to fall in saline-treated sedentary mice, an effect that was restored by training exercise, at 12 weeks. However, the effects of OVX or exercise were not observed for the groups that received any of the ergogenic extracts, according to the analysis at 12 weeks (Supplementary Figure S3A). At 16 weeks, the combination of exercise and TLU greatly improved the resistance to fatigue in OVX animals, whereas the treatment with *E. longifolia* plus exercise significantly diminished this parameter (Supplementary Figure S3B). For males, no significant differences regarding the resistance to fall in the rotarod equipment were detected at 12 (Supplementary Figure S3C) or 16 weeks (Supplementary Figure S3D).

**Figure 5:**
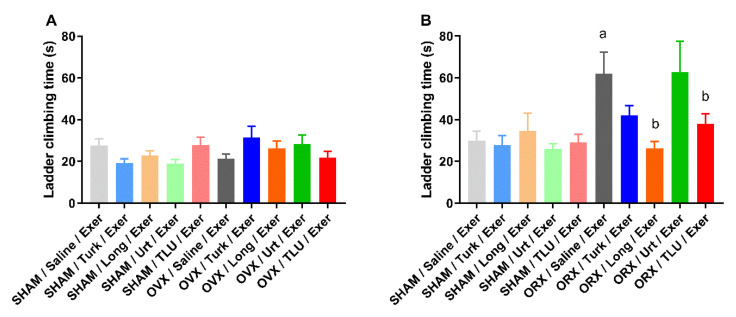
Total time for ladder climbing (s) assessed at 16 weeks, in females (**A**) and males (**B**). SHAM, sham-operated; OVX, ovariectomized; ORX, orchiectomized; Exer, Exercise; Turk, Ajuga turkestanica; Long, Eurycoma longifolia; Urt, Urtica dioica; TLU, the association of the three extracts. Results are the mean ± SEM of 10 to 12 animals per group. ^a^ *p* < 0.05 significantly different compared with the sham-operated group treated with saline. ^b^ *p* < 0.05 significantly different compared with the operated group treated with saline (one-way ANOVA followed by Sidak post hoc test).

As an additional measurement of resistance, the total time spent for ladder climbing was measured in the groups submitted to resistance exercise, at 16 weeks (i.e., at the end of training and treatment protocols). The climbing time did not significantly differ when comparing SHAM and OVX females, regardless of supplementation with any extracts (Figure 5A). For males, castration significantly increased the total climbing time, and this effect was prevented by supplementation with *E. longifolia* extract or TLU, with partial effects for *A. turkestanica* extract. Nonetheless, the phytotherapics did not significantly modify the time to complete the task in SHAM males (Figure 5B).

**Figure 6:**
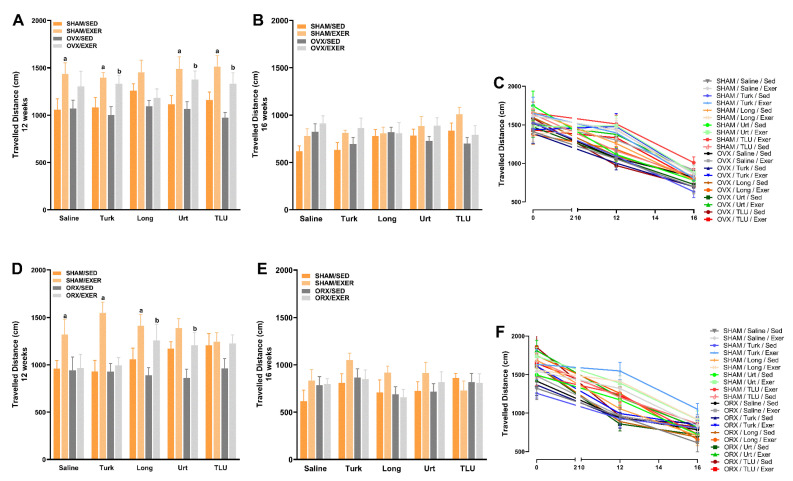
Assessment of spontaneous locomotor activity measured as the travelled distance for 5 min, at 12 and 16 weeks after castration in female ((**A**) and (**B**), respectively) and male mice ((**D**) and (**E**), respectively). Time-course for the travelled distance measured at three different periods: before castration, 12 and 16 weeks after castration, in female (**C**) and male mice (**F**). SHAM, sham-operated; OVX, ovariectomized; ORX, orchiectomized; Sed, sedentary; Exer, Exercise; Turk, Ajuga turkestanica; Long, Eurycoma longifolia; Urt, Urtica dioica; TLU, the association of the three extracts. Results are the mean ± SEM of 10 to 12 animals per group. ^a^ *p* < 0.05 significantly different compared with the sham-operated group. ^b^ *p* < 0.05 significantly different compared with the castrated group (two-way ANOVA followed by Uncorrected Fisher’s LSD).

As for the voluntary locomotion, the exercise training led to an overall increase of travelled distance in SHAM females (Figure 6A) and males (Figure 6B), according to the evaluation at 12 weeks. For OVX mice, the combination of resistance exercise plus *A. turkestanica*, or *U. dioica*, or TLU supplementation, significantly increased the travelled distance. The climbing exercise failed to significantly alter the travelled distance of saline- or *E. longifolia*-treated OVX mice in 12 weeks (Figure 6A). In males, resistance exercise plus supplementation with *E. longifolia*, or *U. dioica* extract enhanced the travelled distance of ORX animals. Oppositely, exercise failed to improve the travelled distance in saline- or *A. turkestanica*-treated ORX groups, with partial effects for TLU, as seen in 12 weeks (Figure 6D). Females (Figure 6C) or males (Figure 6F) displayed a time-related reduction of travelled distance from 12 to 16 weeks, without any significant effects for castration, exercise, or herbal supplementation at 16 weeks (Figure 6B,E).

**Figure 7:**
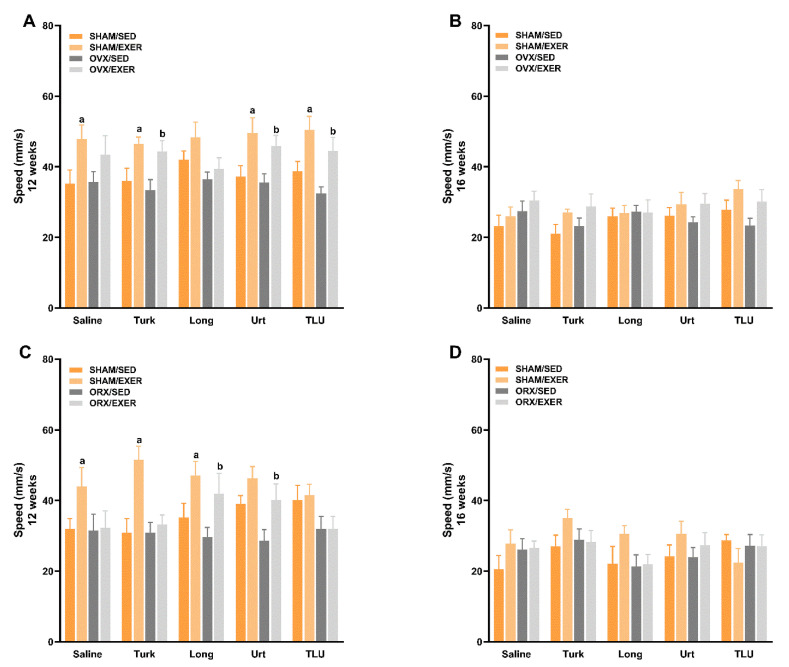
Assessment of spontaneous locomotor activity measured as the animal speed for 5 min, at 12 and 16 weeks after castration in female ((**A**) and (**B**), respectively) and male mice ((**C**) and (**D**), respectively). SHAM, sham-operated; OVX, ovariectomized; ORX, orchiectomized; Sed, sedentary; Exer, Exercise; Turk, Ajuga turkestanica; Long, Eurycoma longifolia; Urt, Urtica dioica; TLU, the association of the three extracts. Results are the mean ± SEM of 10 to 12 animals per group. ^a^ *p* < 0.05 significantly different compared with the sham-operated group. ^b^ *p* < 0.05 significantly different compared to the operated group (two-way ANOVA followed by Uncorrected Fisher’s LSD).

The analysis of speed in the open-field arena revealed general positive effects for exercise training in SHAM female (Figure 7A) or male mice (Figure 7C), as monitored at 12 weeks. In OVX animals, the association of exercise with *A. turkestanica*, or *U. dioica*, or TLU extracts significantly enhanced the speed time of females at 12 weeks (Figure 7A). At the same time-point, ORX males showed an increased speed, in groups that had been submitted to exercise training plus *E. longifolia*, or *U. dioica* extract (Figure 7C). At 16 weeks, no significant difference was observed among the experimental groups, for females (Figure 7B) or males (Figure 7D).

Females (Supplementary Figure S4C) or males (Supplementary Figure S4F) presented a time-related reduction of activity time, independent on the experimental group, from 12 to 16 weeks. This parameter was not significantly modified by OVX or ORX, regardless of exercise or herbal supplementation, at 12 or 16 weeks, for females (Supplementary Figure S4A,B) or males (Supplementary Figure S4D,E), respectively. The combination of resistance exercise plus *A. turkestanica* extract significantly increased the activity time of SHAM male mice at 12 weeks (Supplementary Figure S4D).

### 3.7. Skeletal Muscle Alterations after Exercise and Treatment with Anabolic Extracts

**Figure 8:**
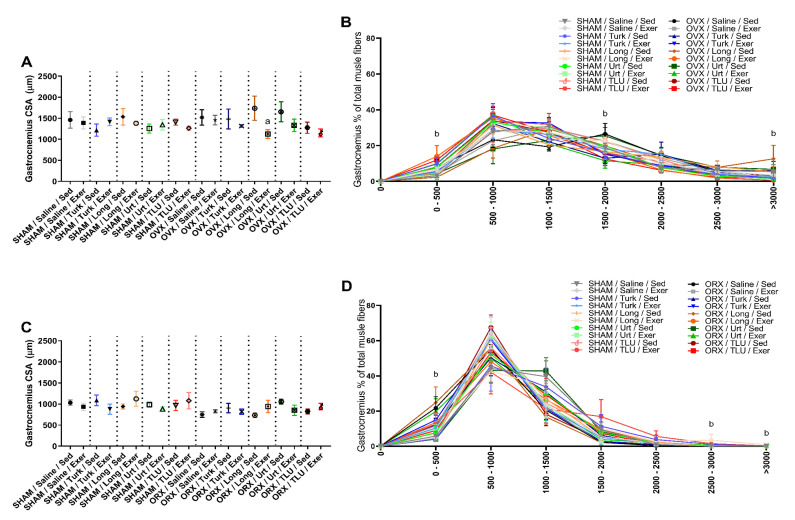
Average of gastrocnemius cross-sectional area of female (**A**) and male mice (**C**) castrated and sham-operated in the different treatment groups. The gastrocnemius fiber size frequency distribution of female (**B**) and male mice (**D**) castrated and sham-operated in the different treatment groups. SHAM, sham-operated; OVX, ovariectomized; ORX, orchiectomized; Sed, sedentary; Exer, Exercise; Turk, Ajuga turkestanica; Long, Eurycoma longifolia; Urt, Urtica dioica; TLU, the association of the three extracts. Results are the mean ± SEM of 5 animals per group. ^a^ *p* < 0.05 significantly different compared to respective sedentary treatment. ^b^ *p* < 0.05 when comparing the groups in each area range. (One-way ANOVA followed by Sidak post hoc test.)

**Figure 9:**
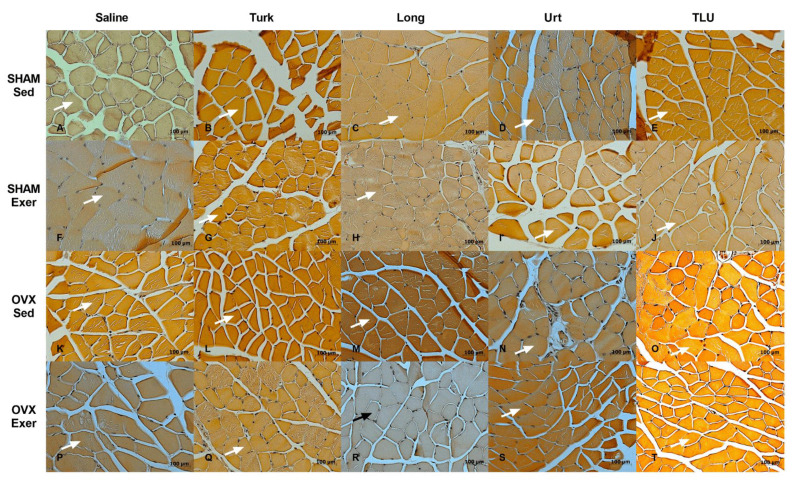
Representative histological images of gastrocnemius of sham-operated mice, sedentary or submitted to exercise, treated with saline (**A**,**F**), Turk 50 mg/kg (**B**,**G**), Long 200 mg/kg (**C**,**H**), Urt 50 mg/kg (**D**,**I**) or the combination of the three extracts TLU (**E**,**J**). Representative histological images of gastrocnemius of ovariectomized mice, sedentary or submitted to exercise, treated with saline (**K**,**P**), Turk 50 mg/kg (**L**,**Q**), Long 200 mg/kg (**M**,**R**), Urt 50 mg/kg (**N**,**S**) and combination of three extracts TLU (**O**,**T**). White arrow indicates gastrocnemius muscle fiber and black arrow indicates gastrocnemius muscle fiber in the group that showed a significant difference of cross-sectional areas (CSA). Turk, *Ajuga turkestanica*; Long, *Eurycoma longifolia*; Urt, *Urtica dioica*; TLU, the association of the three extracts.

Gastrocnemius sections from the different experimental groups were evaluated regarding the cross-sectional areas and the fiber size frequency distribution. In females, only the exercise protocol combined with *E. longifolia* extract treatment caused a significant reduction of cross-sectional area of gastrocnemius muscle in OVX mice. For the other groups, no significant differences were observed for this parameter (Figure 8A). The experimental interventions (i.e., exercise and/or herbal extracts) caused significant alterations of the fiber size frequency distribution. The fibers with 0–500 µm^2^ were significantly higher in OVX mice submitted to exercise plus *E. longifolia* extract treatment, when compared with the respective sedentary group. Sedentary OVX mice treated with *A. turkestanica* extract, or OVX mice submitted to exercise plus *U. dioica* or TLU supplementation showed a reduction in the amount of fibers with 1500–2000 µm^2^, when compared with saline-treated sedentary OVX animals. As for the frequency size >3000 µm^2^, there was a significant higher number of fibers in sedentary OVX mice that had been treated with *E. longifolia* (Figure 8B). Representative histological images of gastrocnemius muscle in different female groups are shown in Figure 9A–T.

**Figure 10:**
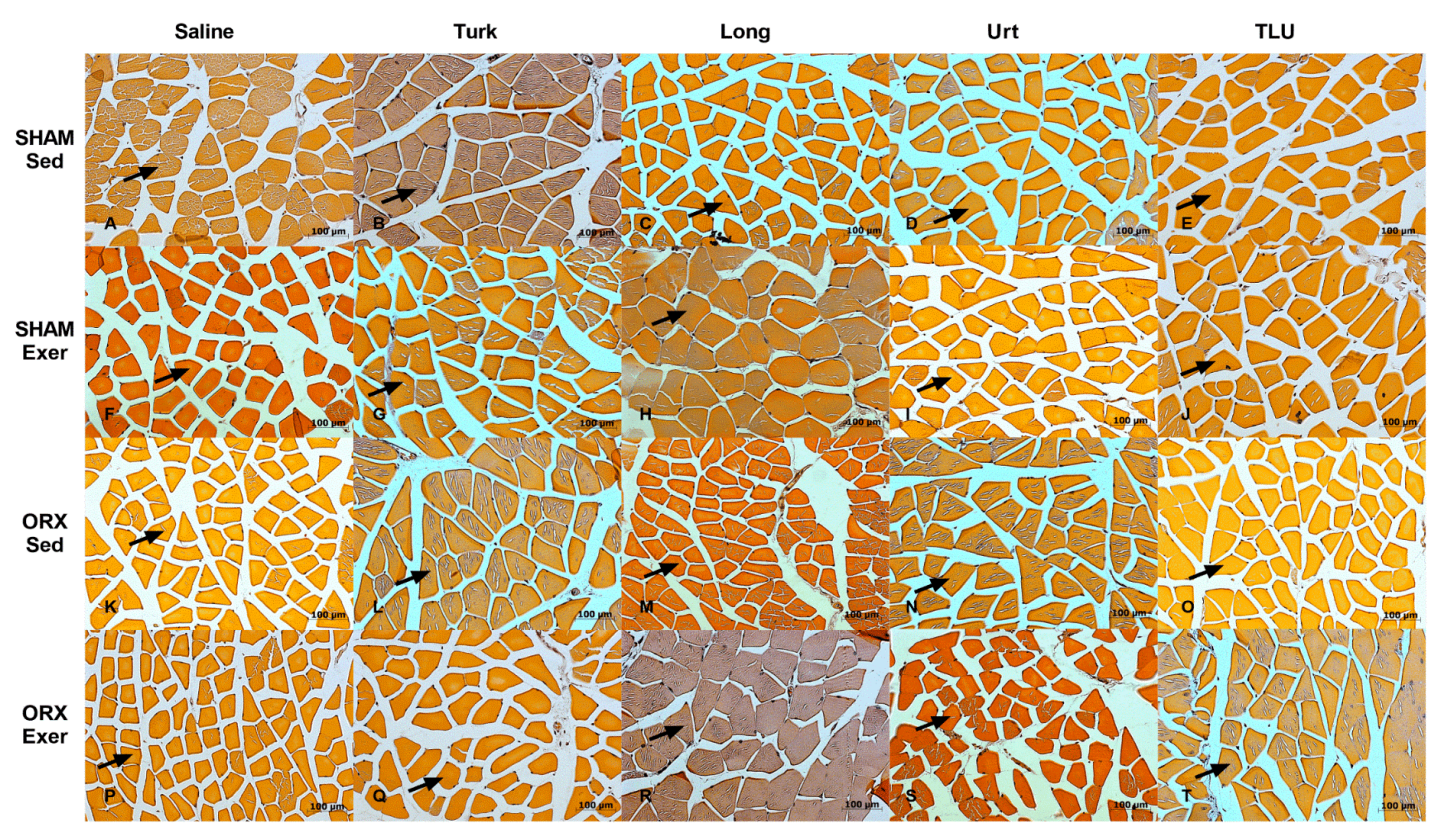
Representative histological images of gastrocnemius of sham-operated mice, sedentary or submitted to exercise, treated with saline (**A**,**F**), Turk 50 mg/kg (**B**,**G**), Long 200 mg/kg (**C**,**H**), Urt 50 mg/kg (**D**,**I**) or the combination of the three extracts TLU (**E**,**J**). Representative histological images of gastrocnemius of orchiectomized mice, sedentary or submitted to exercise, treated with saline (**K**,**P**), Turk 50 mg/kg (**L**,**Q**), Long 200 mg/kg (**M**,**R**), Urt 50 mg/kg (**N**,**S**) and combination of three extracts TLU (**O**,**T**). Black arrow indicates gastrocnemius muscle fiber. Turk, Ajuga turkestanica; Long, Eurycoma longifolia; Urt, Urtica dioica; TLU, the association of the three extracts.

In males, there were no significant differences regarding the gastrocnemius cross-sectional areas, when comparing SHAM and castrated animals, despite exercise or herbal supplementation (Figure 8C). Concerning the fiber size frequency distribution, ORX mice showed a significantly higher number of fibers with 0 to 500 µm^2^. SHAM males that had been trained for climbing and were treated with *E. longifolia* extract showed a significant increase of fibers with 2500–3000 and >3000 µm^2^, in comparison with saline-treated sedentary SHAM males (Figure 8D). The Figure 10 provides representative histological images of gastrocnemius muscle from the 20 experimental groups composed by males.

### 3.8. Histological Evaluation of Adipose Tissue

**Figure 11:**
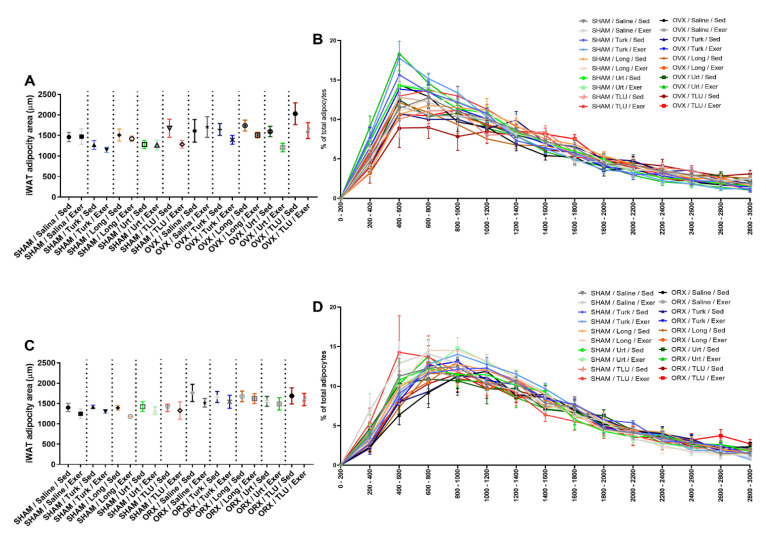
Average adipocyte cross-sectional area from inguinal white adipose tissue of female (**A**) and male mice (**C**) castrated or sham-operated in the different treatment groups. Adipocyte relative frequency area from inguinal white adipose tissue of female (**B**) and male mice (**D**), castrated and sham-operated, in the different treatment groups. SHAM, sham-operated; OVX, ovariectomized; ORX, orchiectomized; Sed, sedentary; Exer, Exercise; Turk, *Ajuga turkestanica*; Long, *Eurycoma longifolia*; Urt, *Urtica dioica*; TLU, the association of the three extracts. Results are the mean ± SEM of *n* = 5 to 7 animals per group (One-way ANOVA followed by Sidak post hoc test).

**Figure 12:**
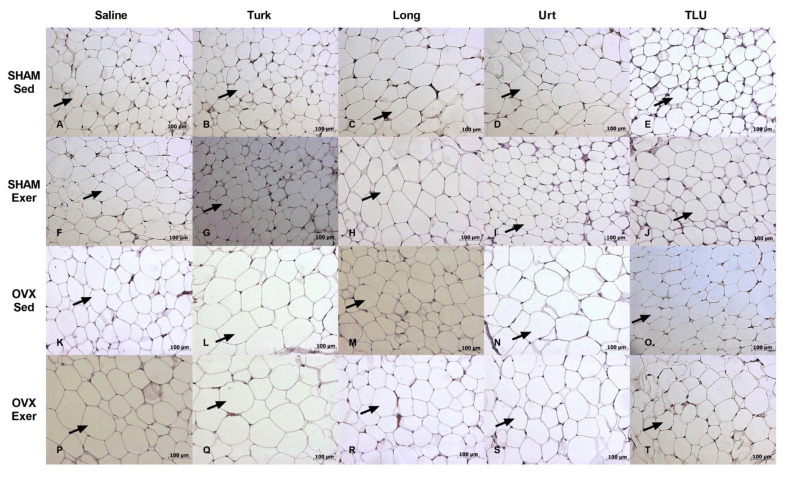
Representative histological images of adipocytes of inguinal white adipose tissue of sham-operated mice, sedentary or submitted to exercise, treated with saline (**A**,**F**), Turk 50 mg/kg (**B**,**G**), Long 200 mg/kg (**C**,**H**), Urt 50 mg/kg (**D**,**I**) or the combination of the three extracts TLU (**E**,**J**). Representative histological images of adipocytes inguinal white adipose tissue of ovariectomized mice, sedentary or submitted to exercise, treated with saline (**K**,**P**), Turk 50 mg/kg (**L**,**Q**), Long 200 mg/kg (**M**,**R**), Urt 50 mg/kg (**N**,**S**) and combination of three extracts TLU (**O**,**T**). Black arrow indicates adipocyte cell. Turk, *Ajuga turkestanica*; Long, *Eurycoma longifolia*; Urt, *Urtica dioica*; TLU, the association of the three extracts.

**Figure 13:**
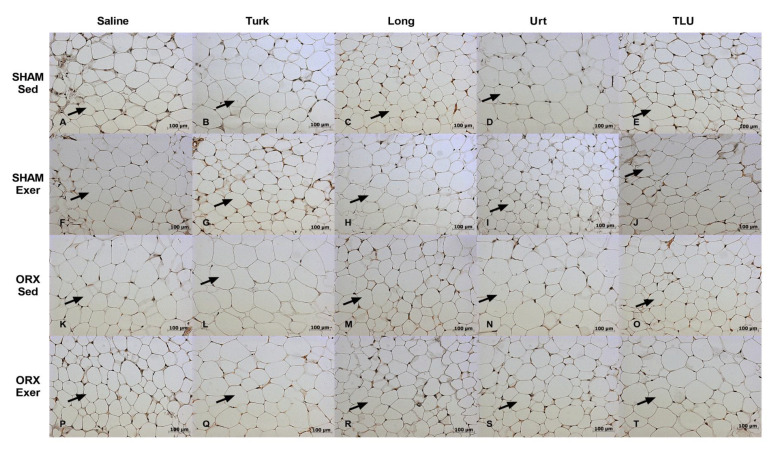
Representative histological images of adipocytes inguinal white adipose tissue of sham-operated mice, sedentary or submitted to exercise, treated with saline (**A**,**F**), Turk 50 mg/kg (**B**,**G**), Long 200 mg/kg (**C**,**H**), Urt 50 mg/kg (**D**,**I**) or the combination of the three extracts TLU (**E**,**J**). Representative histological images of adipocytes inguinal white adipose tissue of orchiectomized mice, sedentary or submitted to exercise, treated with saline (**K**,**P**), Turk 50 mg/kg (**L**,**Q**), Long 200 mg/kg (**M**,**R**), Urt 50 mg/kg (**N**,**S**) and combination of three extracts TLU (**O**,**T**). Black arrow indicates adipocyte cell. Turk, *Ajuga turkestanica*; Long, *Eurycoma longifolia*; Urt, *Urtica dioica*; TLU, the association of the three extracts.

To further evaluate the effects of training exercise and ergogenic extracts on adipose tissue of SHAM and castrated animals, a histological evaluation of iWAT was carried out. The assessment of average size of adipocytes did not show any significant difference when SHAM and OVX females were compared, despite a partial reduction of adipocyte diameter in mice submitted to exercise plus treatments with isolated or combined extracts (Figure 11A). Moreover, no significant differences were observed among the experimental groups, when the frequency size of adipocyte areas were evaluated separately, in either SHAM or OVX mice (Figure 11B). Representative histological images of all 20 experimental groups composed by females are depicted in Figure 12A–T. For males, there were no differences of total adipocyte areas when comparing SHAM with ORX mice, sedentary or submitted to exercise, that had been treated with saline or herbal extracts (Figure 11C). A comparison of the frequency size of adipocytes from the same experimental groups did not show any significant difference (Figure 11D). Histological images of iWAT of male mice (SHAM or ORX) are shown in Figure 13A–T.

### 3.9. Evaluation of Changes in Weights of Kidney, Liver, Brain and Bone

The weight of liver and kidneys was assessed as an indicative of possible toxicity of herbal extracts, or as a consequence of castration or resistance exercise. The training exercise led to a significant reduction of kidney wet weight, in comparison with saline-treated sedentary SHAM females. A significant decrease of kidney weight was also observed in sedentary OVX mice that received *A. turkestanica* extract, or in OVX mice submitted to exercise plus treatment with one of the extracts: *A. turkestanica*, *E. longifolia* or *U. dioica*, dosed isolated. The liver weight was increased in OVX mice that received *A. turkestanica* as supplementation, irrespective of climbing exercise. Brain and femur bone weights were also assessed, but no significant differences were detected when comparing the different female experimental groups (Supplementary Table S1). For males, a significant reduction of kidney and liver weights was observed in saline-treated sedentary ORX mice, when compared with SHAM-matched controls. A trend for a decrease of liver and kidney wet weights was seen in every ORX experimental group, independent on the exercise or treatment. As for femur bones and brain, no significant differences were seen regarding the male groups (Supplementary Table S2).

### 3.10. Biochemical and Inflammatory Parameters

In this part of the study, only saline- or TLU-treated animals distributed in the different experimental groups regarding castration and/or exercise were tested. Castrated females showed an elevation of total and non-HDL cholesterol serum levels. These alterations returned to values seen in saline-treated sedentary SHAM controls, when OVX mice were submitted to resistance exercise, regardless of treatment with TLU. The supplementation with TLU displayed a similar effect in sedentary OVX mice, restoring the total and non-HDL cholesterol levels. The serum levels of triglycerides, TGO or TGP did not significantly differ among the experimental groups composed by females (Table 3). In males, serum cholesterol (total, HDL or non-HDL), triglycerides, TGO or TGP did not present significant differences when comparing SHAM and ORX mice, sedentary or submitted to training exercise, despite the treatment with phytotherapics (Table 4). The pro-inflammatory IL-1β and TNF or the anti-inflammatory IL-10 cytokines were not detected in the gastrocnemius muscle of any experimental group of females or males (Table 3 and Table 4).

## 4. Discussion

In this study, we investigated the anabolic effects of *A. turkestanica*, *E. Longifolia*, and *U. dioica* extracts, administered for two months along with a protocol of resistance exercise, in castrated female and male mice. Hormonal balance is an important factor for maintaining homeostasis, and endocrine alterations can lead to disease development. Indeed, OVX and ORX have been used as rodent models for inducing sarcopenia, due to reduced levels of estrogen and testosterone, respectively [16,39,40,41,42]. In our study, according to that described previously in the literature, OVX led to an increase in body weight gain [63,64]. Even so, ORX promoted a reduction in body weight, accompanied by a decline in muscle mass and accumulation of subcutaneous fat [65,66]. Regarding the tested herbal extracts and the combination of exercise, the main findings show that: (i) the supplementation with *A. turkestanica* associated with exercise recovered the locomotor activity in OVX animals, while in sham females it showed benefits on muscle strength. (ii) In male ORX animals, *A. turkestanica* improved the muscle strength regardless of exercise, whereas the association of the extract with exercise reduced the time to ladder climbing. (iii) *E. longifolia* associated with exercise showed benefits regarding body composition, with an increase in the frequency of larger muscle fibers and a marked reduction in inguinal fat in ORX mice. Additionally, it increased the muscle strength, and the distance and speed covered, besides a reduction in the time to climb the ladder. (iv) The association of *U. dioica* with exercise reduced the final weight in OVX mice. It also increased the travelled distance and speed. (v) In ORX animals, the supplementation with *U. dioica* associated with exercise increased the percentage of muscle mass over total weight, with a reduction in inguinal adiposity. An increase in locomotor activity was also observed. (vi) TLU combined with exercise, in OVX animals, increased the voluntary locomotor activity and the time until fatigue. Furthermore, there was a reduction in non-HDL cholesterol independent on exercise. (vii) Finally, in ORX animals, TLU plus exercise reduced the time to climb the ladder and increased the muscle strength. Collectively, the present data suggests that the tested ergogenic extracts, mainly when combined with resistance exercise, improved sarcopenia alterations associated with hormonal decline, with different profiles in female and male mice.

The prophylactic or therapeutic benefits of physical activity on sarcopenia has been widely demonstrated in the literature. Several variables in the exercise protocol can interfere with the desired effect, such as type, time, frequency and intensity [67]. As described by Beckwée et al. [8], resistance exercise, performed with the use of weights, has positive results in the management of sarcopenia and has been widely indicated for sarcopenic patients [8]. In animal models, the replication of this type of exercise is complex; however, some studies proposed the use of ladder climbing associated with draping of weights in the tail, as a good alternative to reproduce resistance exercise in rodents [67]. Some studies described hypertrophy of the soleus plantaris extensor digitorum longus (EDL) and anterior tibialis muscles, considering their high recruitment in the climbing movement [68]. In our study, the resistance training exercise did not induce muscle hypertrophy in female or male SHAM mice, according to the evaluation of gastrocnemius, tibial, or soleus muscles. Corroborating our data, it was demonstrated that ladder climbing exercise led to hypertrophy of the flexor hallucis longus (FHL), without any effects on gastrocnemius or soleus muscles [69]. A possible explanation for the absence of hypertrophy is that climbing exercise primarily involves concentric, but not eccentric muscle actions, with slight or no muscle mass gain [69,70]. However, the protocol of exercise adopted by us led to increased travelled distance and speed in either female or male SHAM animals, showing its effectiveness to improve physical function, despite the absence of hypertrophy.

Considering the body composition of castrated animals, climbing exercise triggered a reduction of body weight gain in OVX mice, without changes in skeletal muscle or fat mass. In females, estrogen displays a protective role in muscle integrity and function [40]. The reduction of estrogen levels in OVX mice might preclude the effects of exercise in promoting muscle mass gain, despite the overall reduction in body weight. Accordingly, Bunratsami et al. [71] showed a slight reduction of EDL muscle weight in OVX rats, without any change in gastrocnemius—in this case, only a high dose of estrogen was able to induce muscle hypertrophy. The authors also showed that OVX led to a downregulation of ER-α and ER-β receptors in skeletal muscles [71], reinforcing the relevance of estrogen-related pathways for muscle mass gain. As for ORX males, we observed that climbing exercise potentiated the body weight decrease elicited by castration, partly by diminishing the accumulation of iWAT, without any effects on skeletal muscle weights. It has been suggested that low levels of testosterone might be associated with anabolic resistance in aged mice, impacting the muscle responsiveness to exercise, regardless of the benefits on physical function [72]. In OVX and ORX, the resistance exercise also led to improvements of functional parameters, such as muscle strength, resistance to fatigue and physical activity as seen in SHAM animals, but in this case when combined with the tested anabolic extracts. These details will be discussed for each of the three extracts in separated sections.

As a first approach, we decided to analyze the contents of the active compounds in the lyophilized extracts used in the present study. The presence of ecdysteroids, such as turkesterone and β-sitosterol, as well as eurycomanone and other quassinoids in herbal extracts, have been analyzed by using methanol for sample extraction, as it was used in the present study [73,74,75,76]. The initial screening was performed spectrophotometrically, and the identification of active compounds was carried out by LC-MS, and quantified by LC-UV [77], using HDL-cholesterol as standard [78]. The presence of turkesterone (*m*/*z* 497.0 > 443.0 and *m*/*z* 497.0 > 461.0), eurycomanone (*m*/*z* 391.5 > 251.1 and 391.5 > 279.1), and β-sitosterol (*m*/*z* 397.0 > 81.0 and 397.0 > 95.0) was confirmed by mass spectrometry, in lyophilized extracts of *A. turkestanica*, *E. longifolia*, and *U. dioica*, respectively, based on transitions previously described in literature [79,80,81]. Therefore, the lyophilized extracts used in our study contained the corresponding active compounds as described by the commercial supplier.

High doses of the ecdysteroid 20-HE were able to increase the muscle mass and to diminish fat depots in OVX rats, with beneficial effects on lipid metabolism [82]. In our study, the supplementation with *A. turkestanica* in sedentary OVX mice led to reduction of gastrocnemius weight, with an increased frequency of fibers with a small diameter, contrasting somewhat with literature data. However, hormone therapy in women is not likely related to an improvement in muscle mass, despite an increase in muscle strength after estrogen replacement [83,84]. In fact, in the present study, the administration of *A. turkestanica* potentiated the effects of exercise on physical activity in OVX mice, as indicated by a recovery of travelled distance and speed, what might suggest estrogen-related actions for this extract on muscle function. Our suggestion is reinforced by data showing benefits for *A. turkestanica* extract in SHAM females submitted to training exercise, regarding an increase of grip strength.

About the effects of ecdysteroids in males, it was demonstrated that ecdysterone triggers muscle hypertrophy via the activation of ER-β, without any involvement of androgen receptors [85]. Strikingly, estrogen supplementation was demonstrated to revert disuse-related muscle dystrophy in male rodents [86]. In addition, some of the effects previously attributed to testosterone have been currently linked to estrogens, and there is a positive correlation between estrogen levels and muscle strength preservation in men [87]. In our study, the treatment with *A. turkestanica* extract significantly improved the grip strength of ORX mice, independent on training exercise. Moreover, in ORX mice, the supplementation with *A. turkestanica* extract partially restored the time to perform the ladder climbing task, despite the absence of any effect on wet weight of gastrocnemius, tibial or soleus. From this series of results, it is possible to suggest that *A. turkestanica* extract plus resistance exercise differently impacted the muscle function of OVX and ORX mice, mostly favoring increased physical activity in castrated females, whereas it improved muscle strength and force in ORX males.

The effects of standardized extract of *E. longifolia* have been studied in animals and in humans, displaying a positive modulation on testosterone levels [27,28]. In ORX mice, resistance exercise plus *E. longifolia* extract supplementation showed significantly beneficial effects regarding the body composition, with an increase in the frequency of larger muscle fibers, and a marked reduction of iWAT. Supporting our data, *E. longifolia* extract presented antiadipogenic actions, via reduction of PPARγ and C/EBPα expression in the early stage of preadipocyte differentiation in vitro, an effect that was confirmed in vivo in mice [24]. The combination of exercise with *E. longifolia* supplementation also improved the functional performance of ORX mice, by increasing the grip strength and improving travelled distance and speed, with a marked reduction of time necessary to complete the ladder climbing. In SHAM males, only travelled distance and speed were improved by this strategy. It is reasonable to correlate the present findings with the beneficial effects of *E. longifolia* extract on hypogonadism. Accordingly, the active compounds of *E. longifolia*, named eurypeptide and eurycomanone, were able to modulate the biosynthesis of androgens and to reduce testosterone degradation by aromatase inhibition, respectively [27,28,54,88].

A previous publication showed that treatment with *E. longifolia* herbal extract increased the levels of progesterone and estrogen, with mild effects on bone metabolism and testosterone levels, according to the evaluation of OVX rats [27]. In our study, the supplementation with *E. longifolia* extract led to a significant increase of larger muscle fibers in sedentary OVX mice, whereas it increased the frequency of small fibers in OVX mice that had been submitted to exercise training. As for physical aspects, *E. longifolia* plus exercise failed to improve the travelled distance and speed in OVX females and impaired the resistance to fatigue in the rotarod test. The divergent effects of *E. longifolia* extract in castrated males and females might be partly explained by the sex-related differences concerning the responses of skeletal muscles cells to testosterone, with a higher sensitivity for males [86].

A very few studies have investigated the actions of *U. dioica* extract in physical function, and as far we know, there is no previous investigation regarding its effects in sarcopenia. Indeed, its main application is related to treatment of benign prostatic hyperplasia. In addition, some studies have described benefits for *U. dioica* extract for management of diabetes complications [34,57,89]. More recently, it was demonstrated that supplementation with plain *U. dioica* led to body weight loss in mice that received a high-fat diet, via modulation of genes related to lipid and glucose metabolism [33]. In our study, the association of exercise with *U. dioica* extract significantly reduced the final body weight in OVX mice, and also improved the travelled distance and speed in an open arena. Favorable effects for *U. dioica* plus exercise on physical activity were also observed in SHAM females. Conversely, Namjou and collaborators previously described that consumption of *U. dicoica* extract had clear benefits on lipid profile in OVX rats, but not in SHAM controls [90].

In ORX mice, exercise combined with *U. dioica* was able to significantly increase the muscle weight over total body weight, with a reduction of abdominal adiposity, besides an improvement of locomotor activity. Alternatively, this intervention had minimal effects in SHAM males. It was demonstrated that *U. dioica* extract had inhibitory effects on 5α-reductase, the enzyme responsible for the conversion of testosterone in dihydrotestosterone, increasing the serum testosterone levels, without affecting the prostate weight [34]. The raise of free testosterone might explain the elevation in muscle percentage in ORX mice, that had been submitted to exercise plus *U. dicoica* supplementation.

It is well known that andropause and menopause can impact several organs [91]. Additionally, some plant extracts are known by their toxic effects on these organs [92]. Hence, the wet weights of kidneys, liver, femur bone and brain were determined in the different experimental groups of males and females. It was possible to observe that *A. turkestanica* extract reduced kidney weight, while it increased liver mass, according to assessment of sedentary or trained OVX mice. Noteworthy, protective effects for ecdysteroid compounds have been suggested before in models of liver or kidney toxicity [93]. Indeed, estrogen deficiency has been associated with hepatic steatosis [94], and turkesterone-enriched *A. turkestanica* extract might present favorable effects on this condition. For males, ORX led to a reduction of liver and kidney mass, without any effects for exercise and herbal extracts. A previous publication demonstrated that ORX induced a significant decrease of kidney weight, with a slight reduction of liver mass, in mice receiving a standard chow diet [95], supporting somewhat the present data. Overall, castration, exercise, and herbal supplementation had minor effects on organ weights and on most biochemical markers that had been analyzed, suggesting low toxicity levels.

The association of the three extracts was thought to present complementary mechanisms that could generate greater benefits in reversing sarcopenia, as described in folk medicine. In our study, TLU, in combination with resistance exercise, significantly increased the voluntary locomotor activity, and improved resistance to fatigue in OVX mice. Additionally, TLU supplementation recovered the time necessary for ladder climbing and improved the grip strength in ORX mice. Nonetheless, TLU failed to alter some of the tested body composition parameters and physical function, possibly due to the antagonistic effects of the associated extracts, as discussed beforehand.

To gain further insights into the effects of resistance exercise and TLU in castrated mice, we analyzed the serum lipids, triglycerides, besides the markers of tissue damage, namely TGO and TGP. The results did not show any variation of the analyzed biomarkers, except by an increase of total cholesterol and non-HDL cholesterol in saline-treated sedentary OVX mice. A similar alteration of cholesterol levels has been demonstrated before in OVX rats [96]. Of note, both climbing exercise and TLU supplementation, implemented alone or in combination, restored the total and non-HDL cholesterol to the levels seen in SHAM females counterparts. Based on this result, it is time to infer that exercise combined with TLU herbal therapy might also be useful for management of cardiovascular alterations in menopause, besides improving physical performance.

Either inflammatory cytokines, including TNF and IL-1β, or anti-inflammatory cytokines such as IL-10 have been implicated in sarcopenia-related frailty in humans and rodents [97]. Thus, we investigated whether the experimental interventions tested in the present study might modulate the levels of these cytokines in gastrocnemius. Any of the cytokines were undetectable in SHAM, OVX, or ORX groups, irrespective of exercise or TLU supplementation. It is possible to suggest that gains in physical activity or muscle strength observed for the combined intervention with exercise plus extracts did not rely on the modulation of inflammatory markers in skeletal muscle. Further studies are required to assess other inflammatory markers and different biological matrices.

## 5. Conclusions

**Figure 14:**
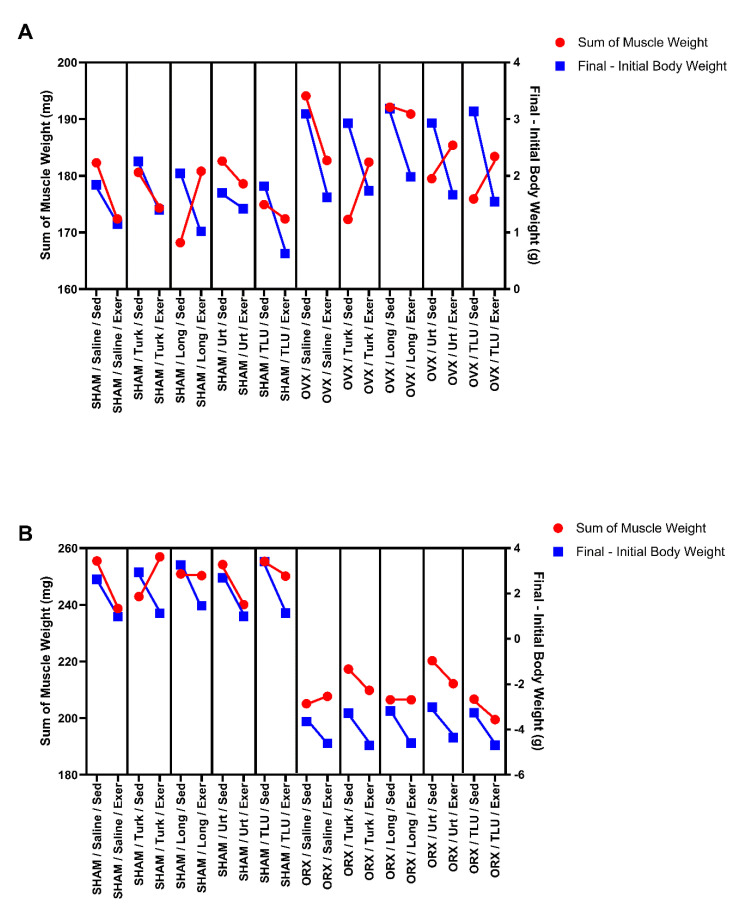
Scheme representing the body weight variation and the summed weight of the three muscles (gastrocnemius, tibial, and soleus) when the animals were submitted to the exercise protocol, in the different treatment groups with: saline, Turk, Long, Urt and TLU, in female sham-operated and ovariectomized mice (**A**) and male sham-operated and orchiectomized mice (**B**). SHAM, sham-operated; OVX, ovariectomized; ORX, orchiectomized; Sed, sedentary; Exer, Exercise; Turk, Ajuga turkestanica; Long, Eurycoma longifolia; Urt, Urtica dioica; TLU, the association of the three extracts.

In this study, using castrated mice, we showed that phytoanabolic extracts of the Eurasian plants *A. turkestanica*, *E. longifolia*, and *U. dioica*, or TLU, differently modulated several parameters related to hormone reduction, such as muscle mass, adiposity, muscle strength, fatigue, general locomotor activity, and lipid profile. These effects were evident when isolated or associated extracts were supplemented in combination with a training exercise protocol. In Figure 14, we provide a schematic illustration about the effects of the tested interventions on body weight variation and summed weight of skeletal muscles (gastrocnemius, soleus and tibial). In saline-treated SHAM males and females and OVX mice, it is possible to observe a trend for reduction in muscle weight, accompanied by a reduction of body weight gain, when the animals were submitted to the ladder climbing exercise. In ORX males, this profile changed, with an inverse effect for exercise on muscle mass and body weight gain. Concerning the supplementation with herbal extracts, *A. turkestanica*, *U. dioica* or TLU changed the profile of body weight gain and skeletal muscle mass in OVX mice. In ORX mice, all of the tested herbal extracts changed the relation between the two parameters in comparison with saline treatment. For sham females and males, there was a change for *E. longifolia* or *A. turkestanica* extracts, respectively. With this, it is possible to identify the importance of the association of exercise with the administration of the extracts considering the management of sarcopenia in menopause and andropause.

Considering the results presented and discussed, we highlight some important points that we identified in our study. *A. turkestanica*, combined with exercise, has a different impact in muscle function of OVX and ORX mice and appears to show better results in ladder climbing speed and muscle strength in ORX mice compared to OVX mice. When evaluating *E. longifolia* plus exercise, it is possible to correlate the results with beneficial effects on hypogonadism. Few studies have investigated the actions of *U. dioica* extract on physical function and, as far as we know, there is no previous investigation on its effects on sarcopenia. With presented results we could mention that *U. dioica* associated with exercise can have beneficial effects on body composition in castrated males and females. Finally, when the association of the extracts was carried out, interestingly, we identified that some of the positive results of the extracts used isolated in the castrated animals were maintained, while the negative results of some of the extracts were mitigated in the association.

## Figures and Tables

**Table 1:** Combined effects of ergogenic extracts and exercise on body, muscle and adipose tissue weight of sham-operated and ovariectomized mice.

	SHAM										OVX									
	Saline/Sed	Saline/Exer	Turk/Sed	Turk/Exer	Long/Sed	Long/Exer	Urt/Sed	Urt/Exer	TLU/Sed	TLU/Exer	Saline/Sed	Saline/Exer	Turk/Sed	Turk/Exer	Long/Sed	Long/Exer	Urt/Sed	Urt/Exer	TLU/Sed	TLU/Exer
Initial Weight (g)	21.3 ± 0.6	19.8 ± 0.4 ^a^	20.1 ± 0.4	20.7 ± 0.5	20.4 ± 0.3	20.7 ± 0.5	20.5 ± 0.5	20.2 ± 0.6	20.6 ± 0.3	20.5 ± 0.3	20.4 ± 0.5	20.6 ± 0.5	20.1 ± 0.5	20.8 ± 0.5	20.4 ± 0.3	20.8 ± 0.4	20.7 ± 0.3	20.3 ± 0.4	20.2 ± 0.3	20.5 ± 0.4
Final Weight (g)	23.2 ± 0.6	21.0 ± 0.4 ^a^	22.4 ± 0.3	21.5 ± 0.4 ^a^	22.4 ± 0.4	21.7 ± 0.3	22.1 ± 0.2	21.6 ± 0.3	22.4 ± 0.4	21.1 ± 0.4 ^a^	23.5 ± 0.5	22.2 ± 0.5	23.0 ± 0.4	22.6 ± 0.5	23.6 ± 0.3	22.8 ± 0.3	23.1 ± 0.4	21.9 ± 0.5 ^b^	23.4 ± 0.5	22.0 ± 0.4
Final-Initial (g)	1.8 ± 0.3	1.1 ± 0.2	2.3 ± 0.2	1.4 ± 0.4	2.0 ± 0.3	1.0 ± 0.4	1.7 ± 0.4	1.4 ± 0.4	1.8 ± 0.3	0.6 ± 0.3	3.1 ± 0.5 ^a^	1.6 ± 0.5 ^b^	2.9 ± 0.3	1.7 ± 0.5	3.2 ± 0.3	2.0 ± 0.2	2.9 ± 0.3	1.7 ± 0.4 ^b^	3.1 ± 0.4	1.5 ± 0.3 ^b c^
Gastrocnemius (mg)	116.2 ± 4.1	110.7 ± 2.6	113.4 ± 2.8	108.9 ± 3.6	107.4 ± 1.4	113.5 ± 3.5	114.6 ± 2.7	110.1 ± 4.5	110.3 ± 3.6	111.0 ± 2.5	123.5 ± 4.4	116.5 ± 2.1	109.4 ± 2.7 ^b^	117.5 ± 3.3	122.6 ± 1.6	122.9 ± 1.2	116.7 ± 2.8	118.3 ± 2.6	112.4 ± 3.2	117.4 ± 3.3
Tibial (mg)	60.4 ± 2.0	56.6 ± 2.1	61.1 ± 1.6	60.3 ± 2.3	60.7 ± 2.3	62.0 ± 2.5	62.4 ± 2.3	62.3 ± 3.1	58.4 ± 2.0	56.4 ± 1.9	65.3 ± 1.5	61.1 ± 2.3	57.0 ± 4.1	59.7 ± 2.4	63.3 ± 1.9	65.9 ± 2.5	58.0 ± 3.0	61.4 ± 1.9	57.7 ± 3.2	61.8 ± 1.5
Soleus (mg)	5.7 ± 0.5	5.1 ± 0.5	5.6 ± 0.4	4.9 ± 0.4	4.5 ± 0.2	5.3 ± 0.4	5.7 ± 0.5	6.2 ± 0.8	5.0 ± 0.4	5.1 ± 0.3	5.3 ± 0.5	5.1 ± 0.7	5.9 ± 0.5	5.2 ± 0.3	6.2 ± 0.5	6.2 ± 0.5	4.9 ± 0.3	5.7 ± 0.5	6.1 ± 0.7	5.5 ± 0.4
Sum of Muscle Weight (mg)	182.3 ± 6.0	172.4 ± 4.6	180.6 ± 4.4	174.3 ± 4.9	168.2 ± 5.6	180.8 ± 6.1	182,6 ± 4.6	178.6 ± 7.7	174,9 ± 5.5	172.4 ± 4.0	194.1 ± 5.4	182.7 ± 4.0	172.3 ± 6.4 ^b^	182.4 ± 5.6	192.1 ± 3.2	190.9 ± 5.8	179.5 ± 5.3	185.4 ± 3.8	175.9 ± 5.7	183.4 ± 4.5
Muscle weight over total weight (%)	0.79 ± 0.02	0.82 ± 0.02	0.82 ± 0.02	0.80 ± 0.02	0.75 ± 0.02	0.83 ± 0.02 ^c^	0.82 ± 0.02	0.82 ± 0.03	0.78 ± 0.02	0.82 ± 0.01	0.81 ± 0.02	0.83 ± 0.02	0.74 ± 0.02	0.81 ± 0.02	0.81 ± 0.01	0.83 ± 0.02	0.77 ± 0.02	0.84 ± 0.01	0.75 ± 0.03	0.83 ± 0.01 ^c^
iBAT (mg)	168.0 ± 11.9	182.1 ± 13.6	145.3 ± 16.6	145.4 ± 15.1	181.3 ± 10.7	133.0 ± 16.3	167.1 ± 14.6	151.3 ± 18.3	197.4 ± 15.7	161.3 ± 10.8	172.0 ± 17.4	178.5 ± 22.1	213.2 ± 26.1	188.4 ± 12.7	197.5 ± 28.6	157.5 ± 22.9	154.2 ± 28.8	139.6 ± 21.3	214.6 ± 16.1	179.2 ± 23.3
iWAT (mg)	118.7 ± 8.2	111.2 ± 7.8	107.4 ± 8.9	99.7 ± 8.2	111.5 ± 6.8	112.6 ± 6.6	106.3 ± 5.1	111.4 ± 8.6	144.6 ± 5.6	120.9 ± 5.0	143.5 ± 12.5	118.9 ± 11.8	144.4 ± 13.1	121.0 ± 11.4	139.5 ± 12.3	114.1 ± 8.7	129.2 ± 12.5	115.7 ± 9.1	163.1 ± 15.3	142.2 ± 6.1
iWAT over total weight (%)	0.51 ± 0.03	0.53 ± 0.03	0.48 ± 0.03	0.46 ± 0.03	0.50 ± 0.03	0.52 ± 0.03	0.48 ± 0.03	0.51 ± 0.04	0.65 ± 0.03	0.57 ± 0.02	0.61 ± 0.05	0.53 ± 0.04	0.62 ± 0.05	0.53 ± 0.04	0.59 ± 0.05	0.50 ± 0.03	0.056 ± 0.05	0.53 ± 0.03	0.69 ± 0.06	0.65 ± 0.02

SHAM, sham-operated; OVX, ovariectomized; Sed, sedentary; Exer, Exercise; Turk, Ajuga turkestanica; Long, Eurycoma longifolia; Urt, Urtica dioica; TLU, the association of the three extracts. Results are the mean ± SEM of 10 to 12 animals per group. ^a^ *p* < 0.05 significantly different compared with the sham-operated group treated with saline. ^b^ *p* < 0.05 significantly different compared with castrated mice treated with saline. ^c^ *p* < 0.05 significantly different comparing exercise to the respective sedentary group. One-way ANOVA followed by Sidak post hoc test.

**Table 2:** Combined effects of ergogenic extracts and exercise on body, muscle and adipose tissue weight of sham-operated and orchiectomized mice.

	SHAM										ORX									
	Saline/Sed	Saline/Exer	Turk/Sed	Turk/Exer	Long/Sed	Long/Exer	Urt/Sed	Urt/Exer	TLU/Sed	TLU/Exer	Saline/Sed	Saline/Exer	Turk/Sed	Turk/Exer	Long/Sed	Long/Exer	Urt/Sed	Urt/Exer	TLU/Sed	TLU/Exer
Initial Weight (g)	27.9 ± 0.5	27.6 ± 0.6	26.8 ± 0.8	27.6 ± 0.5	27.5 ± 0.6	27.9 ± 0.9	27.3 ± 0.4	27.3 ± 0.6	26.8 ± 0.7	28.4 ± 0.5	28.3 ± 0.6	28.5 ± 0.6	27.5 ± 0.4	28.1 ± 0.4	27.5 ± 0.5	27.9 ± 0.4	27.7 ± 0.4	27.6 ± 0.6	27.7 ± 0.5	27.8 ± 0.7
Final Weight (g)	29.5 ± 0.8	28.6 ± 0.8	29.7 ± 0.9	28.8 ± 0.6	30.8 ± 0.5	29.4 ± 1.0	30.0 ± 0.6	28.3 ± 0.7	30.2 ± 0.8	29.6 ± 0.7	24.6 ± 0.6	23.9 ± 0.4	24.6 ± 0.6	23.4 ± 0.3	24.4 ± 0.6	23.3 ± 0.3	24.6 ± 0.3	23.2 ± 0.4	24.41 ± 0.5	23.1 ± 0.4
Final-Initial (g)	2.6 ± 0.3	1.0 ± 0.3 ^a^	2.9 ± 0.4	1.1 ± 0.4 ^a c^	3.3 ± 0.4	1.5 ± 0.3 ^c^	2.7 ± 0.5	1.0 ± 0.2 ^a c^	3.4 ± 0.4	1.1 ± 0.3 ^a c^	−3.7 ± 0.3 ^a^	−4.61 ± 0.5	−3.28 ± 0.3	−4.7 ± 0.2 ^b^	−3.2 ± 0.5	−4.6 ± 0.3 ^c^	−3.0 ± 0.3	−4.36 ± 0.4	−3.3 ± 0.3	−4.7 ± 0.3 ^c^
Gastrocnemius (mg)	159.0 ± 3.0	150.1 ± 4.2	154.2 ± 4.2	158.9 ± 3.7	154.9 ± 1.3	158.0 ± 4.2	155.7 ± 1.6	150.3 ± 3.5	162.2 ± 3.3	157.3 ± 3.4	129.7 ± 2.4 ^a^	132.1 ± 1.8	138.1 ± 2.7	130.9 ± 2.8	129.5 ± 2.0	130.6 ± 3.2	137.5 ± 1.4	134.0 ± 2.8	132.4 ± 2.9	126.1 ± 3.3
Tibial (mg)	88.2 ± 3.1	81.0 ± 1.3	83.5 ± 3.1	84.0 ± 2.9	86.0 ± 2.4	84.8 ± 3.9	90.1 ± 2.7	82.9 ± 2.6	85.5 ± 2.0	84.8 ± 3.2	69.4 ± 2.4 ^a^	68.5 ± 2.7	72.40 ± 3.2	72.7 ± 1.8	70.6 ± 1.9	69.3 ± 2.1	76.2 ± 3.1	72.0 ± 2.1	68,0 ± 2.7	67.1 ± 2.0
Soleus (mg)	8.4 ± 0.6	7.6 ± 0.5	7.3 ± 0.3	8.3 ± 0.5	7.3 ± 0.3	7.6 ± 0.6	8.5 ± 0.5	6.9 ± 0.6	7.6 ± 0.5	7.9 ± 0.5	6.0 ± 0.4 ^a^	7.1 ± 0.5	6.9 ± 0.5	6.2 ± 0.3	6.5 ± 0.5	6.6 ± 0.3	6.6 ± 0.4	6.2 ± 0.4	6.3 ± 0.4	6.3 ± 0.3
Sum of Muscle Weight (mg)	255.5 ± 5.3	238.7 ± 5.6	242.9 ± 7.7	256.9 ± 2.8	250.9 ± 3.9	250.3 ± 8.2	254.2 ± 4.3	240.1 ± 6.1	255.3 ± 4.8	250.1 ± 6.5	205.1 ± 4.5 ^a^	207.7 ± 3.3	217.3 ± 5.7	209.8 ± 3.5	206.5 ± 3.6	206.5 ± 4.8	220.3 ± 3.8	212.2 ± 4.7	206.7 ± 5.3	199.5 ± 4.2
Muscle weight over total weight (%)	0.83 ± 0.01	0.85 ± 0.02	0.80 ± 0.01	0.87 ± 0.01 ^c^	0.81 ± 0.01	0.84 ± 0.02	0.85 ± 0.02	0.85 ± 0.02	0.83 ± 0.01	0.84 ± 0.01	0.85 ± 0.01	0.87 ± 0.02	0.89 ± 0.01	0.90 ± 0.02	0.85 ± 0.02	0.89 ± 0.01	0.88 ± 0.01	0.91 ± 0.01 ^b^	0.85 ± 0.02	0.87 ± 0.01
iBAT (mg)	178.5 ± 5.8	184.1 ± 10.0	188.0 ± 10.0	185.6 ± 7.1	189.4 ± 9.8	171.5 ± 10.6	185.3 ± 6.9	184.1 ± 8.2	185.7 ± 8.1	180.2 ± 10.0	179.5 ± 10.9	174.6 ± 8.1	186.1 ± 12.3	190.6 ± 10.2	183.4 ± 13.5	170.9 ± 7.7	193.7 ± 12.1	164.9 ± 10.4	177.5 ± 9.9	179.5 ± 5.6
iWAT (mg)	111.1 ± 3.86	103.5 ± 11.6	118.7 ± 8.8	106.2 ± 8.9	125.8 ± 6.9	101.5 ± 6.3	106.2 ± 9.1	107.9 ± 6.1	127.5 ± 8.4	113.9 ± 9.4	159.9 ± 9.3 ^a^	127.9 ± 7.1	161.0 ± 13.1	125.7 ± 8.2	169.2 ± 16.6	128.8 ± 8.2 ^c^	149.9 ± 8.3	122.3 ± 9.4 ^b^	148.2 ± 8.3	130.8 ± 6.5
iWAT over total weight (%)	0.34 ± 0.02	0.37 ± 0.04	0.40 ± 0.03	0.37 ± 0.03	0.41 ± 0.02	0.35 ± 0.02	0.36 ± 0.03	0.39 ± 0.03	0.42 ± 0.03	0.39 ± 0.03	0.65 ± 0.03 ^a^	0.54 ± 0.03 ^b^	0.65 ± 0.04	0.54 ± 0.03	0.67 ± 0.06	0.58 ± 0.04	0.63 ± 0.03	0.51 ± 0.03 ^b^	0.61 ± 0.03	0.57 ± 0.03

SHAM, sham-operated; ORX, orchiectomized; Sed, sedentary; Exer, Exercise; Turk, Ajuga turkestanica; Long, Eurycoma longifolia; Urt, Urtica dioica; TLU, the association of the three extracts. Results are the mean ± SEM of 10 to 12 animals per group. ^a^ *p* < 0.05 significantly different compared with the sham-operated group treated with saline. ^b^ *p* < 0.05 significantly different compared with castrated mice treated with saline. ^c^ *p* < 0.05 significantly different comparing exercise to the respective sedentary group. One-way ANOVA followed by Sidak post hoc test.

**Table 3:** Combined effects of ergogenic extracts and exercise on biochemical and inflammatory parameters sham-operated and ovariectomized mice.

	SHAM				OVX			
	Saline/Sed	Saline/Exer	TLU/Sed	TLU/Exer	Saline/Sed	Saline/Exer	TLU/Sed	TLU/Exer
Cholesterol (mg/dL)	76.7 ± 8.2	87.7 ± 5.2	79.2 ± 4.4	83.3 ± 9.3	100.0 ± 6.7	85.12 ± 11.16	89.1 ± 3.2	87.1 ± 4.9
HDL (mg/dL)	51.5 ± 5.2	56.3 ± 4.3	48.7 ± 2.8	58.99 ± 2.1	59.0 ± 3.8	58.2 ± 5.7	57.5 ± 3.0	55.3 ± 3.8
Non-HDL (mg/dL)	30.4 ± 4.5	31.5 ± 2.3	30.5 ± 2.1	36.9 ± 3.6	41.0 ± 3.2 ^a^	35.1 ± 2.2	31.6 ± 2.2	31.8 ± 1.8
Triglycerides (mg/DL)	67.4 ± 7.3	66.1 ± 2.2	62.2 ± 6.1	74.9 ± 5.2	74.2 ± 5.7	70.8 ± 7.5	66.0 ± 8.2	70.4 ± 5.8
TGO (U/L)	56.5 ± 14.0	61.9 ± 13.8	48.9 ± 7.9	53.6 ± 9.5	60.9 ± 12.3	63.8 ± 7.8	44.4 ± 10.4	54.8 ±13.8
TGP (U/L)	32.6 ± 3.0	58.1 ± 17.6	38.3 ± 8.2	45.3 ± 10.2	57.1 ± 5.2	39.8 ± 11.8	31.2 ± 6.7	21.6 ± 18.7
IL-1β	ND	ND	ND	ND	ND	ND	ND	ND
TNF	ND	ND	ND	ND	ND	ND	ND	ND
IL-10	ND	ND	ND	ND	ND	ND	ND	ND

SHAM, sham-operated; OVX, ovariectomized; Sed, sedentary; Exer, Exercise; Turk, Ajuga turkestanica; Long, Eurycoma longifolia; Urt, Urtica dioica; TLU, the association of the three extracts. Results are the mean ± SEM of 10 to 12 animals per group. ^a^ *p* < 0.05 significantly different compared with the sham-operated group treated with saline. One-way ANOVA followed by Sidak post hoc test.

**Table 4:** Combined effects of ergogenic extracts and exercise on biochemical and inflammatory parameters sham-operated and orchiectomized mice.

	SHAM				ORX			
	Saline/Sed	Saline/Exer	TLU/Sed	TLU/Exer	Saline/Sed	Saline/Exer	TLU/Sed	TLU/Exer
Cholesterol (mg/dL)	103.0 ± 7.0	109.3 ± 11.2	91.9 ± 4.4	89.4 ± 5.2	106.1 ± 8.8	96.2 ± 10.1	99.2 ± 1.9	99.4 ± 6.4
HDL (mg/dL)	69.9 ± 7.5	71.2 ± 5.8	55.0 ± 3.4	61.0 ± 6.3	62.7 ± 5.0	64.0 ± 5.8	64.6 ± 5.0	67.0 ± 4.5
Non-HDL (mg/dL)	33.1 ± 2.2	24.6 ± 2.1	36.9 ± 4.1	28.4 ± 5.6	43.4 ± 6.5	32.3 ± 5.8	34.5 ± 5.5	32.4 ± 4.4
Triglycerides (mg/DL)	103.9 ± 1.5	104.5 ± 5.2	107.4 ± 19.1	102.3 ± 10.6	98.1 ± 12.7	93.74 ± 12.12	112.1 ± 15.3	106.9 ± 13.8
TGO (U/L)	101.2 ± 11.5	78.8 ± 9.1	118.0 ± 10.4	86.8 ± 12.3	90.3 ± 19.8	84.0 ± 14.3	95.2 ± 17.3	64.0 ± 9.5
TGP (U/L)	53.0 ± 10.9	48.5 ± 6.9	35.2 ± 4.3	38.8 ± 4.5	35.33 ± 4.9	37.5 ± 7.1	38.4 ± 9.1	37.6 ± 4.7
IL-1β	ND	ND	ND	ND	ND	ND	ND	ND
TNF	ND	ND	ND	ND	ND	ND	ND	ND
IL-10	ND	ND	ND	ND	ND	ND	ND	ND

SHAM, sham-operated; ORX, orchiectomized; Sed, sedentary; Exer, Exercise; Turk, Ajuga turkestanica; Long, Eurycoma longifolia; Urt, Urtica dioica; TLU, the association of the three extracts. Results are the mean ± SEM of 10 to 12 animals per group. One-way ANOVA followed by Sidak post hoc test.

## Supplementary Materials

The following are available online at https://www.mdpi.com/article/10.3390/nu13041177/s1, Figure S1: Distribution of experimental cohorts, Figure S2: Timeline for main experimental procedures, Figure S3: Evaluation of resistance to fatigue in the Rota-rod apparatus in the different experimental groups, Figure S4: Assessment of spontaneous locomotor activity in the different experimental groups; Table S1: Effects of treatment with ergogenic extracts on the wet weight of kidney, liver, bone and brain of sham-operated or ovariectomized mice, Table S2: Effects of treatment with ergogenic extracts on the wet weight of kidney, liver, bone and brain of sham-operated or orchiectomized mice.
