# Supplementary material for: Combined Effects of Exercise and Phytoanabolic Extracts in Castrated Male and Female Mice
Source: Nutrients. 2021 Apr 2;13(4):1177. doi: 10.3390/nu13041177 (PMC8066446; doi:10.3390/nu13041177)
Supplement: Supplementary file 1 [file nutrients-13-01177-s001.pdf]

A

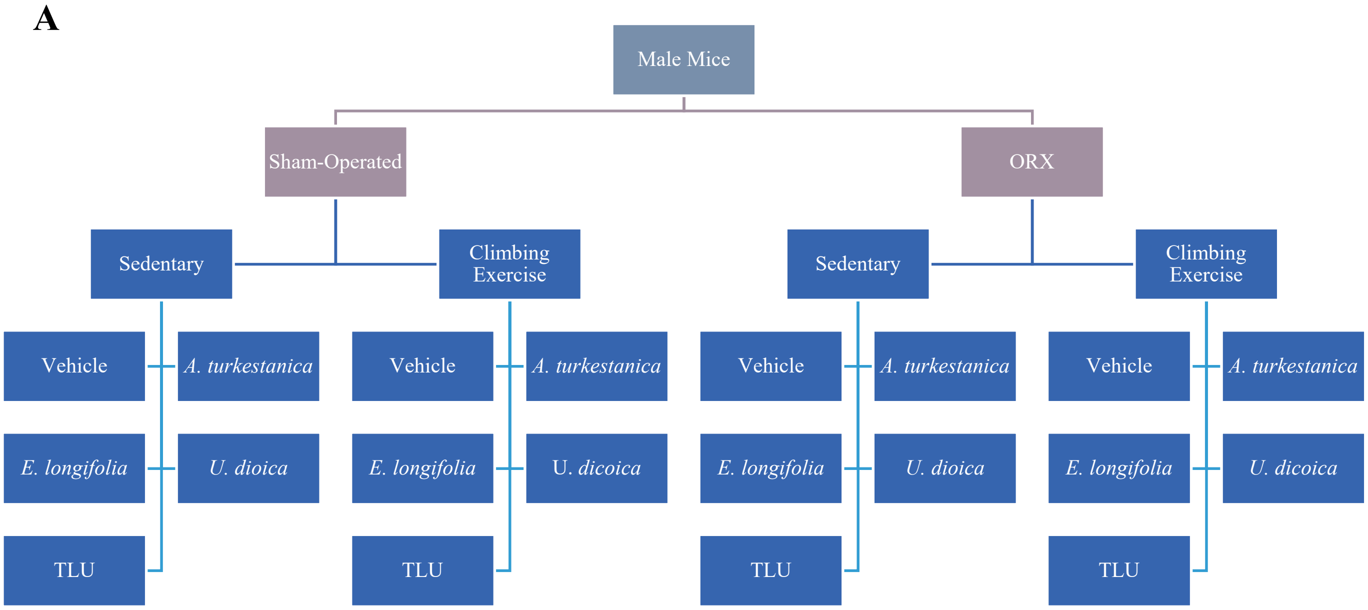

B

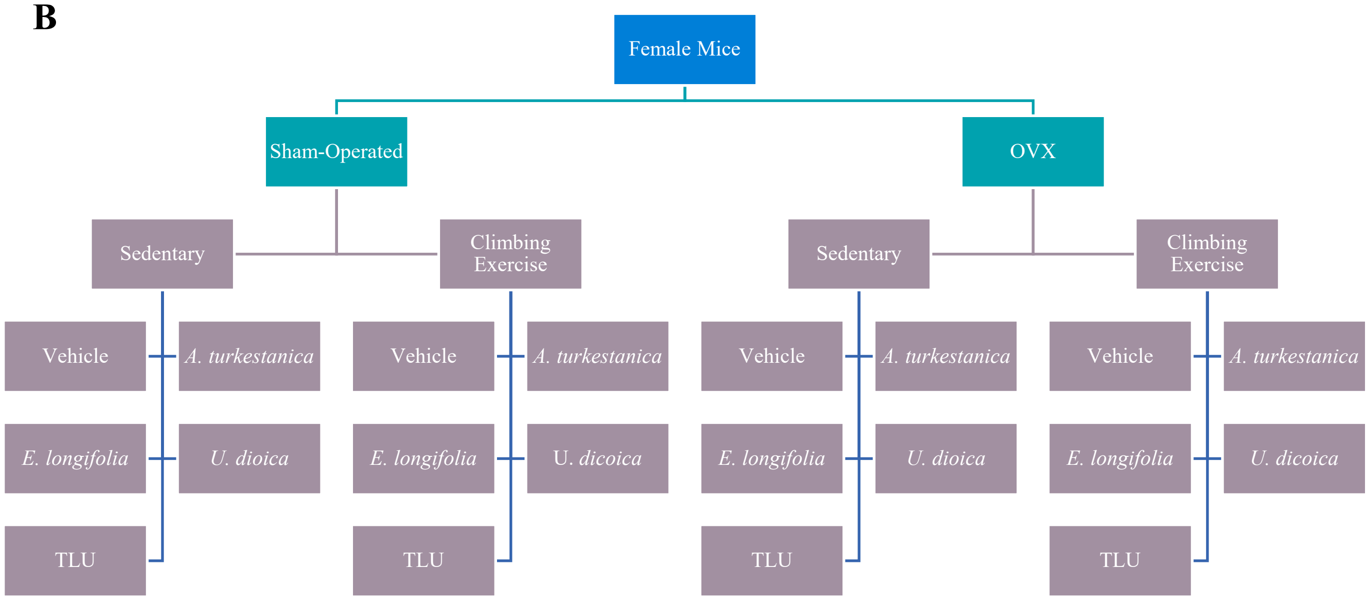

Supplementary Figure 1. Distribution of experimental cohorts. Males (panel A) and females (panel B) were initially divided into two groups: sham-operated and castrated. Subsequently, four additional groups were created from male and female mice, according to physical activity, in sedentary and climbing exercise groups. Finally, these groups were further subdivided according to the treatment with: Vehicle, each one of the lyophilized extracts – *Ajuga turkestanica*, *Euricoma longifolia* or *Urtica dioica* – or the combination of three extracts, named TLU. Twenty experimental cohorts of each sex were evaluated, totaling forty groups.

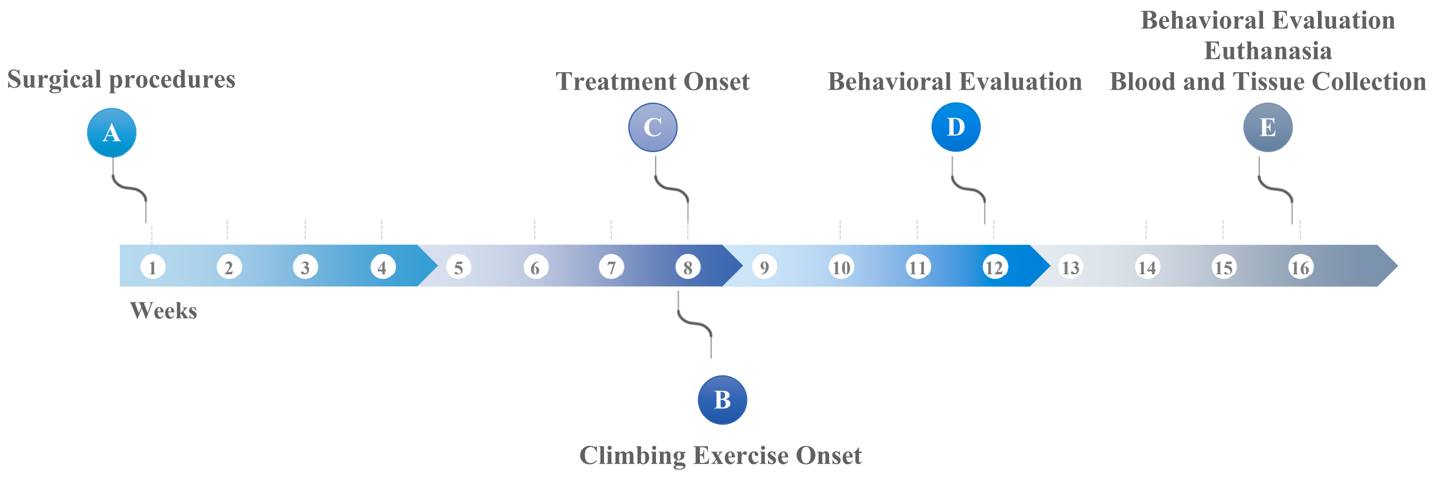

Supplementary Figure 2. Timeline for main experimental procedures. (Panel A) Male and female mice were castrated or sham-operated. (Panel B) Eight weeks later, the animals initiated the resistance exercise protocols for additional eight weeks; half of mice were allocated in the sedentary groups. (Panel C) At eight weeks after surgeries, the treatments with the ergogenic extracts, isolated or combined, began, and continued until the week 16; the control mice received vehicle. (Panels D and E) At 12 and 16 weeks, the animals were evaluated in behavioral tasks, namely locomotor activity in an open-field, motor coordination in Rota-rot, ladder climbing, and grasping test. (Panel E) The animals were euthanized, and blood and tissues were collected for further analysis.

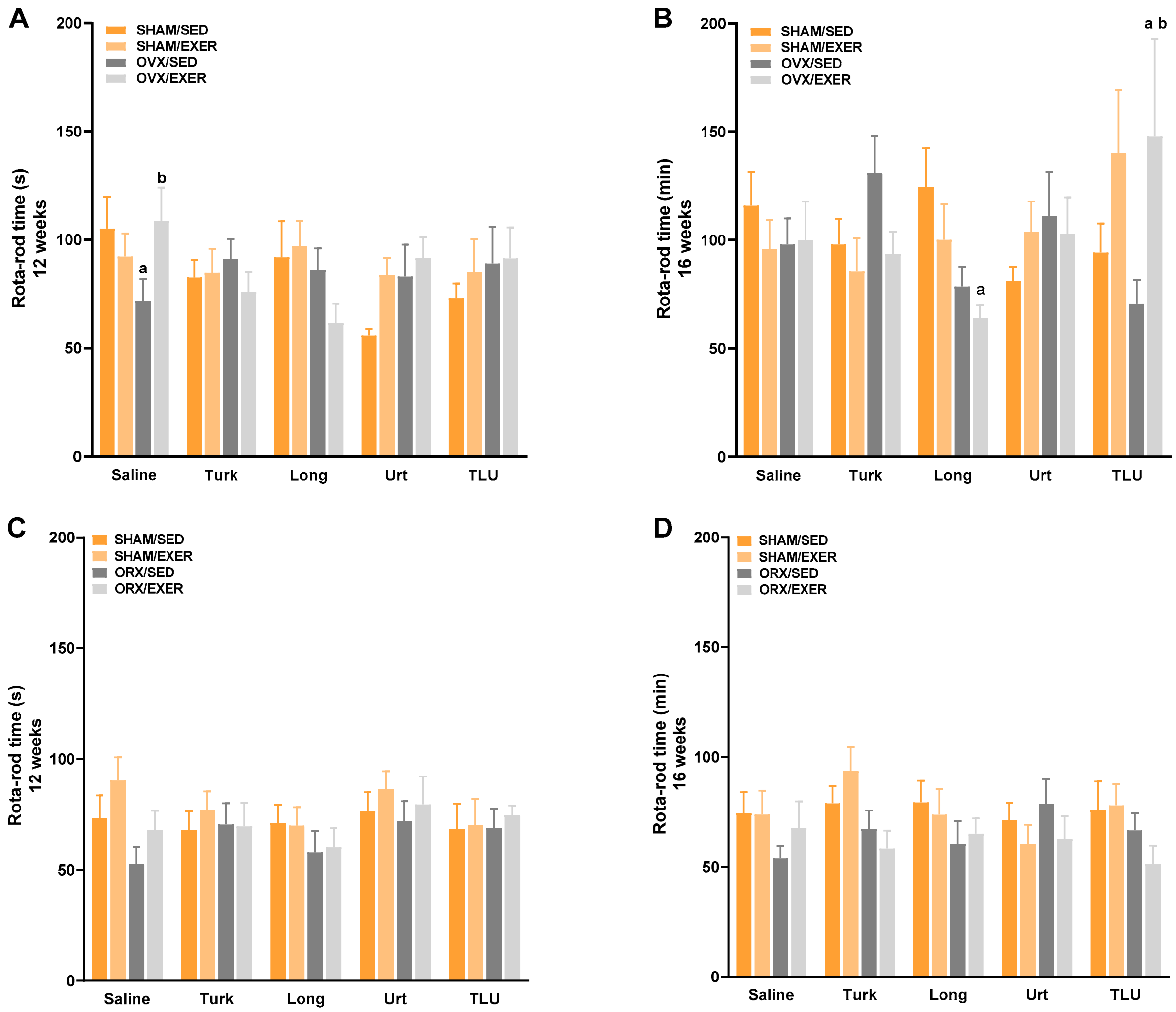

Supplementary Figure 3. Evaluation of resistance to fatigue in the rota-rod apparatus, assessed at 12 and 16 weeks after castration in females (A and B, respectively), or males (C and D, respectively). Results are the mean ± SEM of 10 to 12 animals per group. [a] P<0.05 significantly different in comparison with sham-operated sedentary mice; [b] P<0.05 significantly different compared with sedentary castrated group (Two-way ANOVA followed by Uncorrected Fisher's LSD).

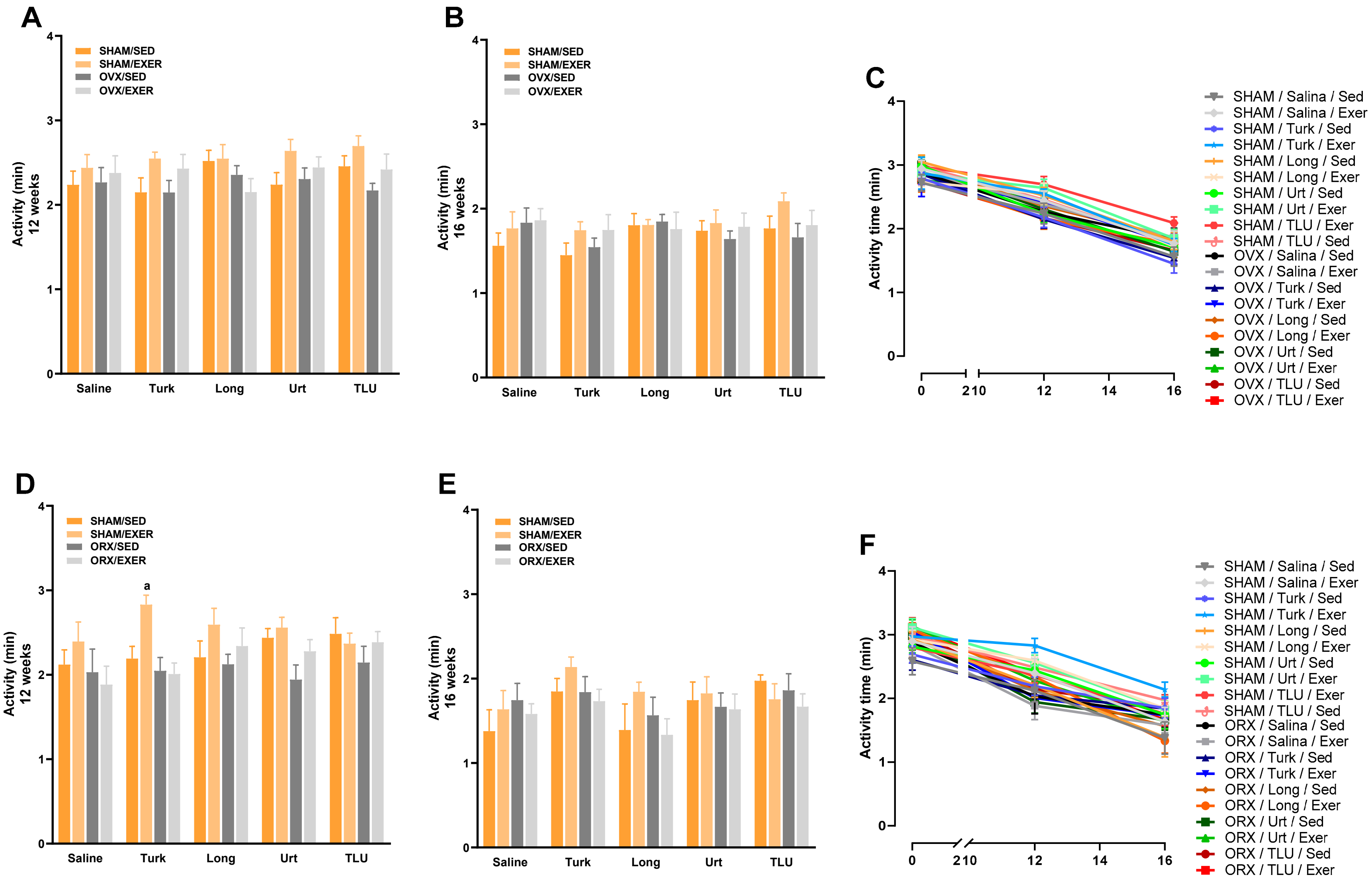
A
B
C
D
E
F
Activity (min) 12 weeks
Activity (min) 16 weeks
Activity time (min)
SHAM/SED
SHAM/EXER
OVX/SED
OVX/EXER
ORX/SED
ORX/EXER
Saline
Turk
Long
Urt
TLU
a
SHAM / Salina / Sed
SHAM / Salina / Exer
SHAM / Turk / Sed
SHAM / Turk / Exer
SHAM / Long / Sed
SHAM / Long / Exer
SHAM / Urt / Sed
SHAM / Urt / Exer
SHAM / TLU / Exer
SHAM / TLU / Sed
OVX / Salina / Sed
OVX / Salina / Exer
OVX / Turk / Sed
OVX / Turk / Exer
OVX / Long / Sed
OVX / Long / Exer
OVX / Urt / Sed
OVX / Urt / Exer
OVX / TLU / Sed
OVX / TLU / Exer
ORX / Salina / Sed
ORX / Salina / Exer
ORX / Turk / Sed
ORX / Turk / Exer
ORX / Long / Sed
ORX / Long / Exer
ORX / Urt / Sed
ORX / Urt / Exer
ORX / TLU / Sed
ORX / TLU / Exer

Supplementary Figure 4. Assessment of spontaneous locomotor activity measured as the acitivty time for 5 min, at 12 and 16 weeks after castration in female (A and B, respectively) and male mice (D and E, respectively). Time-course for activity time from surgical procedures until 16 weeks in female (C) and male mice (F). Results are the mean ± SEM of 10 to 12 animals per group. [a] $P<0.05$ significantly different in comparison with sham-operated sedentary mice (Two-way ANOVA followed by Uncorrected Fisher's LSD).

SupplementaryTable 1. Effects of treatment with ergogenic extracts on the wet weight of kidney, liver, bone and brain of sham-operated or ovariectomized mice.

	SHAM										OVX									
	**Salina / Sed**	**Salina / Exer**	**Turk / Sed**	**Turk / Exer**	**Long / Sed**	**Long / Exer**	**Urt / Sed**	**Urt / Exer**	**TLU / Sed**	**TLU / Exer**	**Salina / Sed**	**Salina / Exer**	**Turk / Sed**	**Turk / Exer**	**Long / Sed**	**Long / Exer**	**Urt / Sed**	**Urt / Exer**	**TLU / Sed**	**TLU / Exer**
**Kidney (mg)**	146.2 ± 7.2	126.6 ± 4.1 [a]	133.1 ± 4.1	138.1 ± 3.5	148.1 ± 8.4	131.4 ± 4.1	127.4 ± 4.9	129.4 ± 4.1	137.2 ± 3.9	128.3 ± 6.6	153.7 ± 8.2	139.0 ± 6.7	130.9 ± 5.3 [b]	128.7 ± 3.5 [b]	133.0 ± 5.9	129.5 ± 3.5 [b]	138.2 ± 6.2	122.2 ± 2.9 [b]	135.2 ± 6.2	136.9 ± 7.5
**Liver (mg)**	999.5 ± 45.7	895.5 ± 33.9	944.0 ± 25.9	945.1 ± 39.2	941.7 ± 54.8	881.6 ± 24.0	957.7 ± 20.0	904.4 ± 43.2	931.5 ± 51.72	936.6 ± 44.6	938.7 ± 46.0	1006.0 ± 40.9	1097.0 ±29.2 [b]	1094.0 ±32.2 [b]	1027.0 ± 33.6	1039.0 ± 21.9	1034.0 ± 37.7	1044.0 ± 33.2	1055.0 ± 63.0	1005.0 ± 30.4
**Bone (mg)**	89.4 ± 6.7	90.1 ± 3.7	93.8 ± 5.6	89.6 ± 5.0	87.6 ± 5.4	88.4 ± 4.4	93.0 ± 5.5	81.2 ± 5.3	87.2 ± 3.2	91.0 ± 2.1	84.6 ± 3.4	86.0 ± 5.3	89.5 ± 5.6	88.2 ± 4.3	89.7 ± 6.8	93.8 ± 8.0	99.8 ± 5.8	93.4 ± 4.6	98.0 ± 2.4	94.0 ± 4.7
**Brain (mg)**	456.0 ± 5.3	437.0 ± 5.8	455.7 ± 6.8	447.0 ± 8.3	457.3 ± 3.5	439.1 ± 7.2	453.9 ± 5.1	450.2 ± 6.2	444.7 ± 5.9	445.1 ± 5.6	452.1 ± 5.9	448.1 ± 6.4	440.4 ± 6.6	449.1 ± 4.3	462.3 ± 5.1	455.0 ± 5.1	454.8 ± 4.8	441.9 ± 7.4	444.3 ± 6.3	447.1 ± 5.1

SHAM, sham-operated; OVX, ovariectomized; Sed, sedentary; Exer, Exercise; Turk, Turkesterone; Long, Eurycoma longifolia; Urt, Urtica dioica; TLU, the association of the three extracts.

Results are the mean ± SEM of 10 to 12 animals per group.

[a] *$P<0,05$ significantly different compared with the sham-operated group treated with saline.*

[b] *$P<0,05$ significantly different compared with castrated mice treated with saline.*

One-way ANOVA followed by Sidak post hoc test.

Supplementary Table 2. Effects of treatment with ergogenic extracts on the wet weight of kidney, liver, bone and brain of sham-operated or orchictomized mice.

	SHAM										ORX									
	**Saline / Sed**	**Saline / Exer**	**Turk / Sed**	**Turk / Exer**	**Long / Sed**	**Long / Exer**	**Urt / Sed**	**Urt / Exer**	**TLU / Sed**	**TLU / Exer**	**Saline / Sed**	**Saline / Exer**	**Turk / Sed**	**Turk / Exer**	**Long / Sed**	**Long / Exer**	**Urt / Sed**	**Urt / Exer**	**TLU / Sed**	**TLU / Exer**
**Kidney (mg)**	178.3 ± 4.7	170.3 ± 9.9	168.4 ± 6.1	165.8 ± 5.9	186.1 ± 7.7	177.9 ± 6.3	188.8 ± 9.2	164.2 ± 5.8	183.6 ± 16.2	180.1 ± 8.7	120.8 ± 5.4 [a]	126.1 ± 5.8	124.6 ± 4.7	116.7 ± 3.9	119.2 ± 3.8	115.4 ± 3.9	122.1 ± 3.8	123.8 ± 6.8	117.1 ± 6.0	120.1 ± 4.2
**Liver (mg)**	1238.0 ± 41.2	1153.0 ± 30.1	1195.0 ± 49.3	1143.0 ± 40.3	1176.0 ± 53.9	1185.0 ± 50.1	1176.0 ± 51.3	1154.0 ± 46.6	1130.0 ± 39.8	1117.0 ± 39.4	1018.0 ± 26.5 [a]	991.0 ± 41.3	912.9 ± 33.6	973.8 ±46.2	1017.0 ± 33.7	989.8 ± 29.5	998.8 ± 38.7	936.5 ± 35.6	1042.0 ± 34.2	1000.0 ± 32.0
**Bone (mg)**	104.1 ± 3.8	103.1 ± 3.1	101.3 ± 3.9	104.3 ± 1.4	99.69 ± 4.0	108.8 ± 3.3	104.2 ± 3.7	100.0 ± 4.3	105.2 ± 3.4	105.2 ± 2.9	98.4 ± 4.0	101.5 ± 3.2	105.5 ± 3.1	96.29 ± 3.2	97.6 ± 4.9	101.5 ± 3.2	94.5 ± 4.7	94.3 ± 3.9	99.1 ± 2.1	100.4 ± 3.5
**Brain (mg)**	450.5 ± 6.7	451.2 ± 5.4	459.3 ± 5.3	456.1 ± 7.8	460.8 ± 3.7	450.2 ± 4.5	455.7 ± 6.8	455.5 ± 4.4	456.9 ± 6.2	463.4 ± 5.6	449.1 ± 6.6	445.9 ± 7.4	453.8 ± 4.3	444.5 ± 5.1	441.8 ± 6.5	447.2 ± 5.1	454.2 ± 2.4	456.5 ± 6.1	444.0 ± 8.4	446.0 ± 3.7

SHAM, sham-operated; ORX, orchiectomized; Sed, sedentary; Exer, Exercise; Turk, Turkesterone; Long, Eurycoma longifolia; Urt, Urtica dioica; TLU, the association of the three extracts.

Results are the mean ± SEM of 10 to 12 animals per group.

[a] *P<0,05 significantly different compared with the sham-operated group treated with saline.*

One-way ANOVA followed by Sidak post hoc test.
